# GEF14 acts as a specific activator of the plant osmotic signaling pathway by controlling ROP6 nanodomain formation

**DOI:** 10.1038/s44319-025-00412-w

**Published:** 2025-03-13

**Authors:** Lucille Gorgues, Marija Smokvarska, Caroline Mercier, Clara P Igisch, Amandine Crabos, Armelle Dongois, Vincent Bayle, Jean-Bernard Fiche, Philippe Nacry, Marcelo Nollmann, Yvon Jaillais, Alexandre Martinière

**Affiliations:** 1 IPSiM Univ Montpellier, CNRS, INRAE, Institut Agro, Montpellier, France; 2 UMR 5200 Membrane Biogenesis Laboratory, CNRS and University of Bordeaux, INRAE Bordeaux, Villenave d’Ornon, France; 3 Laboratoire Reproduction et Développement des Plantes, Université de Lyon, ENS de Lyon, UCB Lyon 1, CNRS, Université de Lyon, ENS de Lyon, UCB Lyon 1, CNRS, INRAE, 69342 Lyon, France; 4 Centre de Biochimie Structurale, Centre National de la Recherche Scientifique Unité Mixte de Recherche 5048, Institut National de la Santé et de la Recherche Médicale U1054, Université de Montpellier, 34090 Montpellier, France

**Keywords:** Rho GTPase, Osmotic Signaling, Auxin, Reactive Oxygene Species, Guanidine Exchange Factor, Membranes & Trafficking, Plant Biology, Skin

## Abstract

During their growth, plants encounter and respond to a variety of environmental signals. However, the mechanisms underlying the integration and specificity of signals remain poorly understood. Rho of Plant (ROP) signaling plays a central role in various processes, including polar cell growth and responses to different stimuli, and relies on stimuli-dependent membrane nanodomains. The effector composition of ROP6 nanodomains varies depending on the signal and may be involved in downstream signal specificity. In this study, we explore how ROP6 signaling is regulated by Guanine nucleotide Exchange Factor (GEF) during osmotic stress. We find that GEF14 is required for osmotically induced ROS accumulation. This isoform acts specifically in response to osmotic stimulation, since it is dispensable for other stimuli. We demonstrate that GEF14 activates ROP6 and controls its clustering in a signal-specific manner. Furthermore, we find that GEF14 relocates from the cytoplasm to clusters at the plasma membrane after osmotic stimulation. Together, our results suggest that a single GEF isoform can encode for signal specificity controlling ROP6 activation, clustering and downstream cellular responses.

## Synopsis

Rho Of Plant 6 (ROP6) acts as hub for several independent signaling processes and relies on stimuli-dependent membrane nanodomain formation. This study reveals that the GEF isoform GEF14 is essential for osmotically induced ROS accumulation and activates ROP6 in a signal-specific manner during osmotic stimulation.

- Rho Of Plant 6 (ROP6) is a hub for membrane signaling.
- Guanine nucleotide Exchange Factor 14 (GEF14) confer osmotic signaling specificity.
- GEF14 regulates ROP6 nanodomains formation during osmotic signaling.

## Abstract (web summary)

Rho Of Plant 6 (ROP6) acts as hub for several independent signaling processes and relies on stimuli-dependent membrane nanodomain formation. This study reveals that the GEF isoform GEF14 is essential for osmotically induced ROS accumulation and activates ROP6 in a signal-specific manner during osmotic stimulation.

## Graphical abstract

**Figure:**
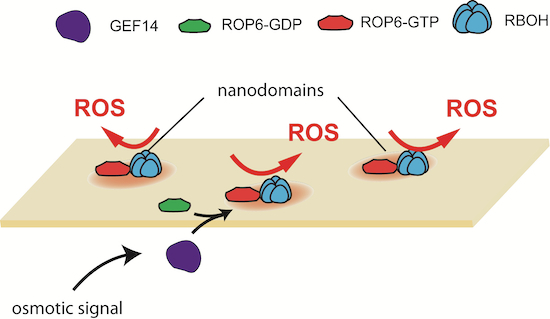

**Figure:**
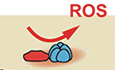

## Introduction

Cell signaling networks produce output decisions to generate developmental and physiological responses. They are generally composed of combinatorial connections of individual signaling pathways. In these structures, nodes or hubs play a critical role for network topology and determine their emergent properties such as ultrasensitivity, bistability, oscillatory behavior or robustness (Azeloglu and Iyengar, 2015). From a molecular point of view, biochemical information is transferred via coupled reactions, which are temporally and spatially controlled. How spatiotemporal parameters, especially within biological membranes, determine signaling network properties is an emerging question in biology.

Biological membranes are composed of a myriad of lipids and proteins. These compounds are not homogeneously distributed in membranes, and are arranged in domains of varying size and composition. Paradoxically, membranes are also fluid structures, allowing the lateral mobility of their constituents through thermal agitation. This property of the membrane is essential for the dynamic distribution of its constituents and thus regulates signaling processes (Jaillais and Ott, 2020; Martinière and Zelazny, 2021).

In plants, the role of membrane dynamic partitioning is particularly important for Rho of Plant (ROP) signaling processes (Feiguelman et al, 2018; Smokvarska et al, 2021; Pan et al, 2023; Platre et al, 2019). Indeed, ROPs can form micrometer-sized domains that are necessary for polar cell growth. This is notably the case for the development of root hairs, pollen tubes or lobes in epidermal pavement cells (PVC) (Feiguelman et al, 2018). This property to form polar domains is a conserved feature even in *Marchentia polymorpha* (Rong et al, 2022). Interestingly, ROPs can also reorganize from a homogeneous distribution in the plasma membrane (PM) into nano-sized domains in response to external stimuli (Platre et al, 2019; Smokvarska et al, 2020). For example, ROP6 forms nanodomains in response to auxin, which are needed for cellular and tissue responses to this phytohormone (Platre et al, 2019; Pan et al, 2020). ROP6 nanodomains formation is also a key factor in triggering osmotic signaling pathways in plant cells (Smokvarska et al, 2020). Consequently, in a given cell, ROP6 nanodomains formation is involved in signal transmission of two different stimuli. While an osmotic signal induces ROP6 nanodomains containing Respiratory Burst Oxidases homologues D and F (RBOHD/F) to catalyze Reactive Oxygen Species (ROS) production, one of the earliest signaling responses to osmotic stimulation, we do not observe ROP6/RBOHD/F nanodomains nor ROS accumulation in response to the phytohormone auxin (Smokvarska et al, 2020). Thus, the effectors present in ROP6 nanodomains seems to differ depending on the nature of the initial upstream stimulus. How such specificity at the membrane is achieved still remains an open question.

ROPs belong to the superclade of Ras/Rho GTPase and work as molecular switches due to their conformational change between active GTP-bound form and inactive GDP-bound form (Wittinghofer and Berken, 2008). The catalytic switch between its active and inactive form is mediated through GEFs that facilitate GDP release and GAP that enhance GTP hydrolysis (Wittinghofer and Berken, 2008). Because they are direct activators of ROP, GEFs were proposed to participate in the establishment of Rho GTPase signaling specificity (Marei and Malliri, 2017). Plants have two major types of GEFs: a plant specific family containing the catalytic domain PRONE and GEF containing the dock homology region 2 (DHR2) (Qiu et al, 2002; Berken et al, 2005; Gu et al, 2006; Basu et al, 2008). SPIKE1 (SPK1) is the unique DHR2-type of GEF in Arabidopsis genome and was demonstrated to act on cytoskeleton regulation and needed for auxin-mediated ROP6 activation (Basu et al, 2008; Lin et al, 2012). Interestingly, ROP6 was also demonstrated to interact with several PRONE-containing GEFs. GEF14 was shown to immunoprecipitate with ROP6 and is needed to activate ROP6 in pavement cells. In addition, GEF4 and GEF10 show a protein-protein interaction with ROP6 by Bimolecular fluorescence complementation and yeast two hybrids (Huang et al, 2013). Those results suggest that several GEFs can interact and potentially regulate ROP6 activation depending of the cellular context and upstream stimulus. But, if some of those isoforms control signal specificity and how GEFs act on ROP6 membrane partitioning remains unexplored.

Here, we show that ROP6 is not only required for osmotic and auxin responses but is also involved in PAMPs and ABA signaling. Thus, our results suggest that ROP6 is acting as a signaling hub, controlling different signaling pathways at the membrane. However, we found that GEF14 acts specifically in the osmotic signaling pathway. Furthermore, we demonstrate that GEF14 can activate ROP6 by controlling ROP6 dynamic and clustering, specifically in response to the osmotic signal.

## Results

### ROP6 is necessary for short-term cell signaling responses after treatment with different stimuli

**Figure 1:**
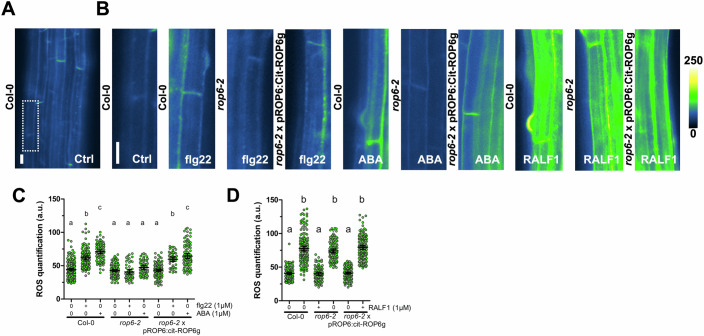
ROP6 is necessary to trigger ROS accumulation in response to certain stimuli. (**A**) Images of dihydroethidium (DHE) stained root where the dash region of interest highlight the epidermal cell. (**B**) DHE stained root epidermal cells of Col-0, *rop6-2* or *rop6-2*xpROP6:cit-ROP6g in presence or absence of flg22 or ABA. (**B**) DHE fluorescence quantification in control condition, after 30 min of 1 µM flg22 or 60 min of 1 µM ABA with the different genetic material. (**C**) Images of dihydroethidium (DHE) stained root cells of Col-0, *rop6-2* or *rop6-2*xpROP6:cit-ROP6g in control condition or in presence of RALF1 peptide. (**D**) DHE fluorescence quantification in control condition or after 15 min of 1 µM of RALF1 treatment with the different genetic material. Mean with Error bars correspond to the 95% confidence interval. According to analysis of variance (ANOVA) followed by a Tukey test, letters indicate significant differences among means (*P* < 0.05). *n* > 26 cells from three to four independent biological replicas. Scale bar, 10 μm. a.u., arbitrary units.

ROP6 mediates short-term response to both auxin (Platre et al, 2019) and osmotic signals ((Smokvarska et al, 2020), Fig. EV1A,B). We tested if its functions could be extended to other signals. To this end, we treated, wild type, *rop6-2* and *rop6-2*xpROP6:cit-ROP6g plants with the bacterial PAMP, flg22 or the phytohormone, ABA that are both known to trigger ROS accumulation in Arabidopsis root cells (Yang et al, 2014; Poncini et al, 2017). DMSO was used as ABA solvent and this treatment shows no effect on ROS accumulation compared to the control condition (Fig. EV1C). As expected, we found that both flg22 and ABA treatment are able to induce ROS accumulation in wild-type plants (Fig. 1A–C). Interestingly, this ROS accumulation is compromised in *rop6-2* and restored in the *rop6-2*xpROP6:cit-ROP6g line (Fig. 1A–C). In addition, we tested the effect of the Rapid Alkanisation Factor 1 (RALF1) since it is also known to induce ROS accumulation in root tips (Yu et al, 2018). Contrary to flg22 and ABA treatment, we found that ROS induced by RALF1 is not mediated through ROP6 (Fig. 1C,D). Those results suggest that in root epidermal cells, ROP6 mediates ROS signaling in response to some, but not all signals.

**Figure EV1:**

ROP6 is needed for osmotically-induced ROS accumulation. (**A**) Images of dihydroethidium (DHE) stained root cells of Col-0, *rop6-2* or *rop6-2*xpROP6:cit-ROP6g in control condition (0 MPa) or after application −0.75 MPa solution. (**B**) DHE fluorescence quantification in control condition, after 15 min treatment with −0.75 MPa solution with the different genetic material. (**C**) DHE fluorescence quantification in control condition or with DMSO. Mean with Error bars correspond to the 95% confidence interval. (**B**) According to analysis of variance (ANOVA) followed by a Tukey test, letters indicate significant differences among means (*P* < 0.05). (**C**) *t*-test, n.s. non-significant difference, *p*-value = 0.089. *n* > 31 cells from three to four independent biological replicas. Scale bar, 10 μm. a.u., arbitrary units.

### GEF14 acts specifically on osmotic signaling

**Figure 2:**
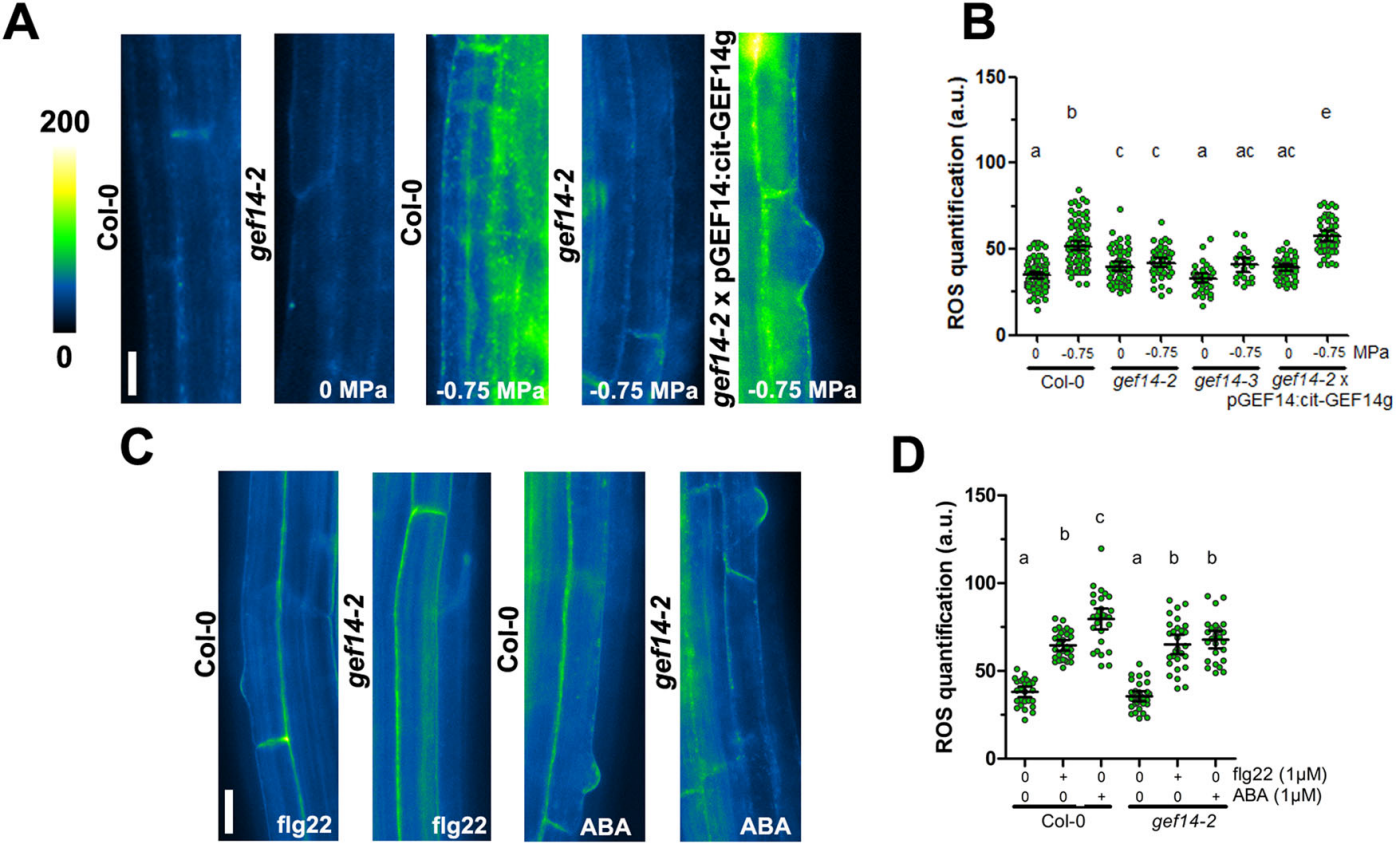
GEF14 is specifically involved in osmotically induced ROS accumulation. (**A**) Images of dihydroethidium (DHE) stained root cells of Col-0, *gef14-2, gef14-3,* or *gef14-2*xpGEF14:cit-GEF14g in control condition (0 MPa) or after application −0.75 MPa solution. (**B**) DHE fluorescence quantification in control condition, after 15 min treatment with −0.75 MPa solution with the different genetic material. (**C**) Images of DHE stained root cells of Col-0 or *gef14-2* in control condition after 30 min of 1 µM flg22 or 60 min of 1 µM ABA and its respective DHE fluorescence quantification (**D**). Mean with Error bars correspond to the 95% confidence interval. According to analysis of variance (ANOVA) followed by a Tukey test, letters indicate significant differences among means (*P* < 0.01). *n* > 26 cells from three to four independent biological replicas. Scale bar, 10 μm. a.u., arbitrary units.

Since 14 GEF isoforms exist in Arabidopsis genome, our previous results prompted us to investigate whether a single signal can mediate a specific ROP6 response through a given GEF. The GEF gene family is known to directly interact with ROP proteins and mediate their conformation changes from GDP (inactive) to GTP (active) bound form. Indeed, GEF14 has recently been shown to interact with ROP6 in Arabidopsis pavement cells (PCV) (Lin et al, 2021; Tang et al, 2021). Moreover, the two knock-out alleles *gef14-2* and *gef14-3* show no osmotically induced ROS accumulation after 15 min of osmotic treatment, complementation line with genomic GEF14 sequence express under its endogenous promoter recovers fully the response (Fig. 2A,B). These results show that GEF14 is necessary to trigger osmotic signaling in Arabidopsis root epidermal cells. Yet, ROP6 was also described to interact with other GEF isoforms, especially GEF4 and 10 (Huang et al, 2013). To determine if other GEFs are involved in osmotic signaling, a series of knockout lines were tested. Neither GEF2, GEF3, GEF4, GEF10 nor GEF11 are needed to osmotically induced ROS accumulation (Fig. EV2A). To rule out any potential redundancy effect, GEF multiple mutants were also tested. Again, only when *GEF14* was absent the osmotically induced ROS accumulation was lost (Fig. EV2B). Then, we wondered if GEF14 acts specifically on osmotic signaling. Therefore, wild type and *gef14-2* plants were incubated either with flg22 or ABA that both induce ROS accumulation in a ROP6-dependent manner (Fig. 1B). Interestingly, *gef14-2* is still able to accumulate ROS after those treatments (Fig. 2C,D). Taken together these results suggest that among the tested GEF isoforms, GEF14 is the unique GEF that is required to sustain the osmotic signaling in root epidermal cells. In addition, we showed that GEF14 is dispensable for the other ROP6-related signaling pathways.

**Figure EV2:**

Characterization of osmotically-induced ROS accumulation in various *GEFs* knock-out lines. (**A**) DHE fluorescence quantification in control condition or after 15 min treatment with −0.75 MPa solution on different single *GEF* knock-out lines or triple and quadruple GEF mutants lines. (**B**) Mean with Error bars correspond to the 95% confidence interval. (**B**) According to analysis of variance (ANOVA) followed by a Tukey test, letters indicate significant differences among means (*P* < 0.05). *n* > 21 cells from three independent biological replicas. a.u. arbitrary units.

### GEF14 and ROP6 expression pattern overlap in root cells and GEF14 formed discrete foci at the PM after an osmotic stimulation co-localized with ROP6

**Figure 3:**
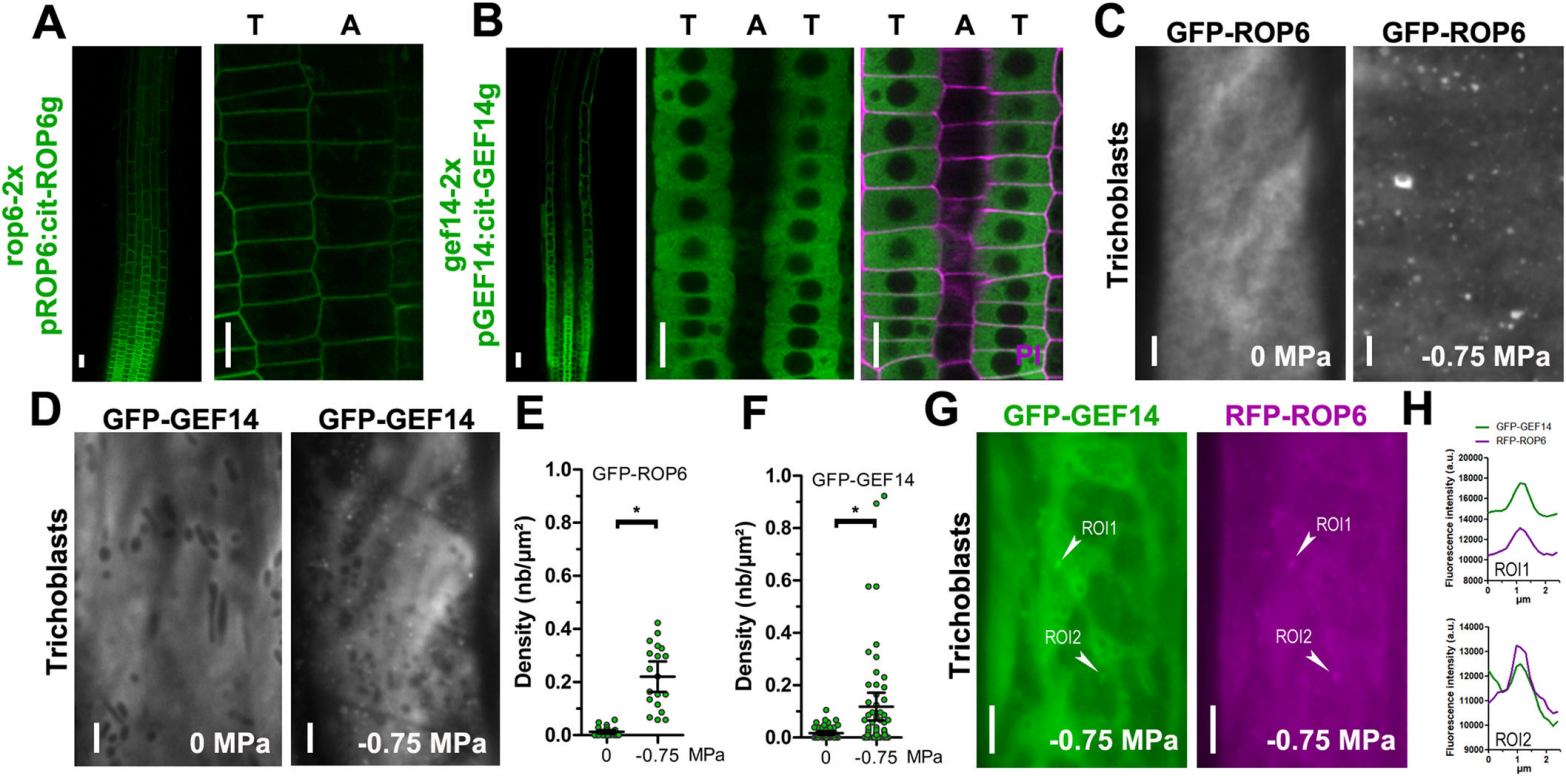
GEF14 is expressed in trichoblasts cells and forms cluster at the PM after osmotic stimulation that co-localized with ROP6. (**A**) Fluorescent signal for *rop6-2*xpROP6:cit-ROP6g expressing plants in root tip with a zoom in on meristematic zone. (**B**) Fluorescent signal for *gef14-2*xpGEF14:cit-GEF14g expressing plants in root tip with a zoom in on the meristematic zone (counter stained with propidium iodide, PI). TIRF images of GFP-ROP6 (**C**) or GFP-GEF14 (**D**) expressing trichoblast cells in control condition or after 15 min treatment with a −0.75 MPa solution. (**E**, **F**) Quantification of cluster density in GFP-ROP6 or GFP-GEF14 expressing trichoblast cells in control condition or after 15 min treatment with a −0.75 MPa solution. (**G**) Dual-color TIRF image of trichoblast cell expressing GFP-GEF14 and RFP-ROP6 after 15 min treatment with a −0.75 MPa solution. (**H**) Pixel intensity plot of GFP-GEF14 (green) and RFP-ROP6 (magenta) across dot in ROI1 and ROI2. Mean with Error bars correspond to the 95% confidence interval. (**E**) *t-test, *p*-value > 0.0001*.* (**F**) *t-test, *p*-value = 0.0003. *n* > 19 cells from three independent biological replicas. Scale bar, 10 μm for (**A**) and (**B**). Scale bar, 3 µm for (**C**), (**D**) and (**G**). a.u., arbitrary units, T, trichoblast cell, A, atrichoblast cell.

ROP6 and GEF14 are both needed to trigger osmotic signaling in root cells. If these two proteins are indeed functionally linked, they should be expressed in the same cells. As previously described, the signal of *rop6-2*xpROP6:cit-ROP6g lines label the epidermal cell in the meristem and also in the elongation zone (Fig. 3A) (Lin et al, 2012; Smokvarska et al, 2020; Marquès-Bueno et al, 2021). At a cellular level, ROP6 was mostly localized at the PM and polarly localized in the root hair initiation domain (RHID) at the onset of root hair bulging (Figs. 3A and EV3A). Similar to cit-ROP6g, *gef14-2*xpGEF14:cit-GEF14g was also expressed in the meristematic and elongation zone, but its expression was restricted to trichoblast cells and GEF14 accumulated in the cytoplasm rather than the plasma membrane (Denninger et al, 2019) (Fig. 3B). Only a small portion of cit-GEF14g localized at the PM at the RHID (Fig. EV3A, arrows). ROP6 and GEF14 both accumulate in the RHID. We thus wondered if the osmotically-induced ROS accumulation described earlier takes rise at this location. Recently, GEF3 was shown to be essential to determine the RHID polarity in trichoblast cells and is subsequently important to relocalize GEF4 and ROP2 to trigger root hair emergence (Denninger et al, 2019). We wonder if a similar mechanism could drive GEF14 RHID localization. Interestingly in *gef3-1*xpGEF14:cit-GEF14g, cit-GEF14g fluorescent signal is lost at the RHID (Fig. EV3B). This shows that in *gef3-1*, GEF14 is not accumulated in RHID. Yet, osmotically-induced ROS accumulation is still happening in *gef3-1* (Fig. EV2A). This result suggests that osmotic signaling mediated by GEF14 is not happening at the RHID. Previously, we showed that ROP6 forms very fast osmotic-dependent nanodomains. The formation of those nanodomains are required to trigger osmotic signaling (Smokvarska et al, 2020). We applied total internal reflection microscopy (TIRF) to lines expressing either GFP-ROP6 or GFP-GEF14. In trichoblast cells, a part of GFP-ROP6 appears in clusters after an osmotic signal (Fig. 3C,E). GFP-GEF14 signal in control condition is uniformly distributed in the cytoplasm, whereas after osmotic stimulation GFP-GEF14 appeared in discrete immobile foci at or close to the plasma membrane (Fig. 3D,F). In addition, dual-color TIRF microscopy shows that GFP-GEF14 and RFP-ROP6 co-localized (Fig. 3G,H). Thus, osmotic stimulation induces GFP-GEF14 relocalisation from the cytoplasm to ROP6-containing nanodomain at the plasma membrane.

**Figure EV3:**

GEF14 RHID localization is controlled by GEF3. (**A**) Fluorescent signal of rop6-2xpROP6:cit-ROP6g and *gef14-2*xpGEF14:cit-GEF14g in root tip (arrows show RHID). (**B**) Close-up view of *gef14-2*xpGEF14:cit-GEF14g signal with a propidium iodide (PI) counter staining. (**C**) Fluorescent signal of *gef3-1*xpGEF14:cit-GEF14g in root tip and its respective ratio (RHID/cytoplasm) quantification (**D**). Mean with Error bars correspond to the 95% confidence interval (**D**) *t*-test, *p*-value < 0.0001. *N* > 27 cells from two independent experiments. White arrow point to the root hair initiation domain (RHID). Scale bar, 10 μm.

### GEF14 is an activator of ROP6

**Figure 4:**
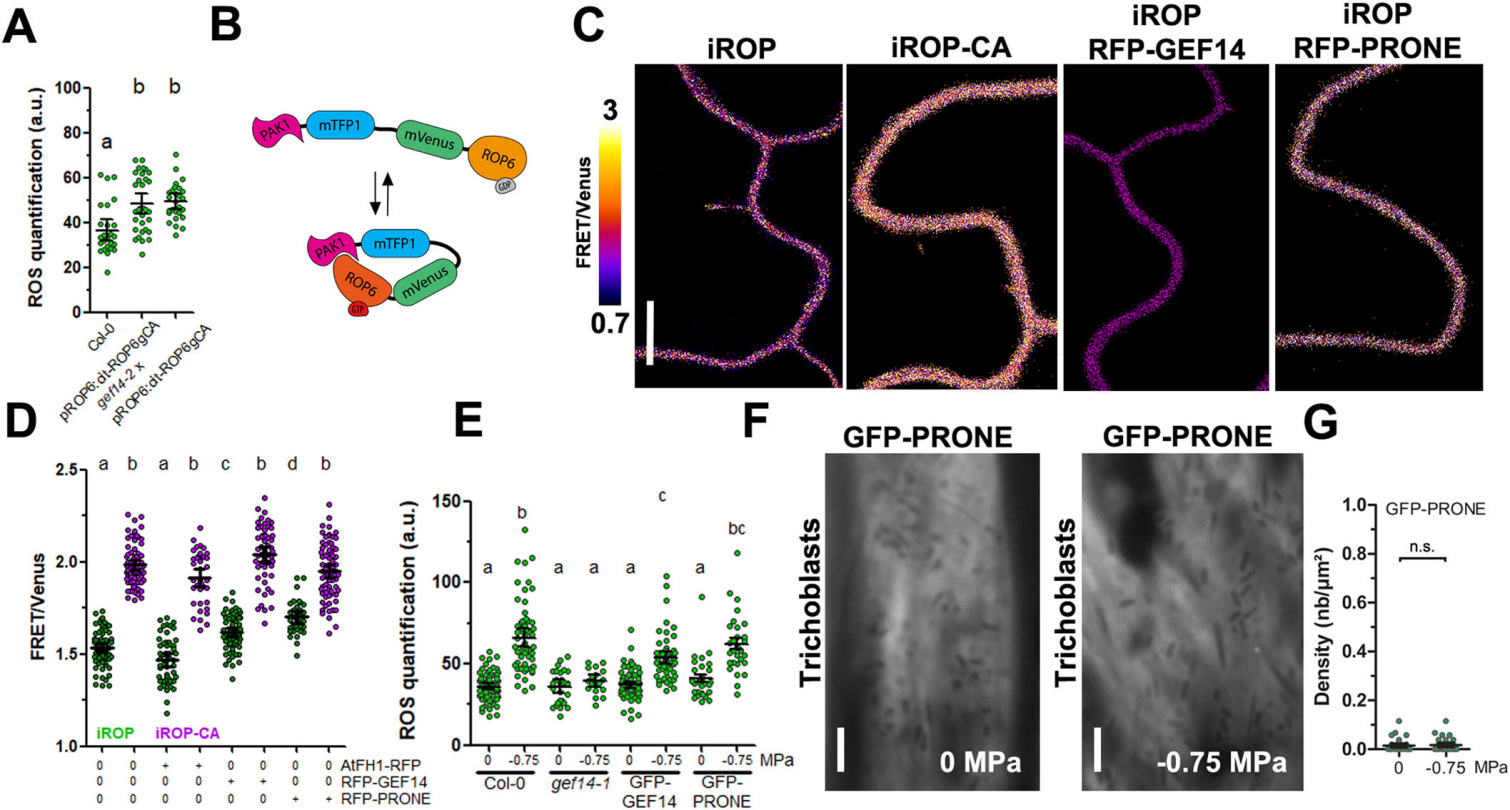
GEF14 is an activator of ROP6. (**A**) DHE quantification of Col-0, pROP6:cit-ROP6gCA (ROP6g containing the constitutive active mutation G15V) and *gef14-2*xpROP6:cit-ROP6gCA. (**B**) Schematic view of iROP sensor. ROP6 GTP-bound form interacts with PAK1, allowing FRET between Venus and mCherry. In contrast, when ROP6 is inactive (in its GDP-bound form) the distance between the two fluorescent proteins increases, thereby diminishing FRET efficiency. (**C**) Ratio images (channel^FRET^/channel^mVenus^) of iROP or iROP-CA where ROP6 contain the constitutive active mutation G15V expressed tobacco cells, with or without RFP-GEF14 or RFP-PRONE. (**D**) Quantification of ratio signal (channel^FRET^/channel^mVenus^) obtained from iROP or iROP-CA expressing tobacco cells in combination with the negative control AtFH1-RFP, RFP-GEF14 or RFP-PRONE. (**E**) DHE quantification of Col-0, *gef14-2*, GFP-GEF14 or GFP-PRONE expressing Arabidopsis root cells in control condition or after 15 min treatment with −0.75 MPa solution. (**F**) TIRF images of GFP-PRONE in control or after a 15 min treatment with −0.75 MPa solution and its respective cluster density quantification (**G**). Mean with Error bars correspond to the 95% confidence interval. (**A**, **D**, **E**) According to analysis of variance (ANOVA) followed by a Tukey test, letters indicate significant differences among means (*P* < 0.05). *n* > 16 cells from three independent biological replicas. (**G**) n.s. non-significant difference, *t*-test, *p*-value = 0.635, *n* > 13 cells from two independent biological replicas. Scale bar, 10 μm. a.u., arbitrary units.

**Figure EV4:**
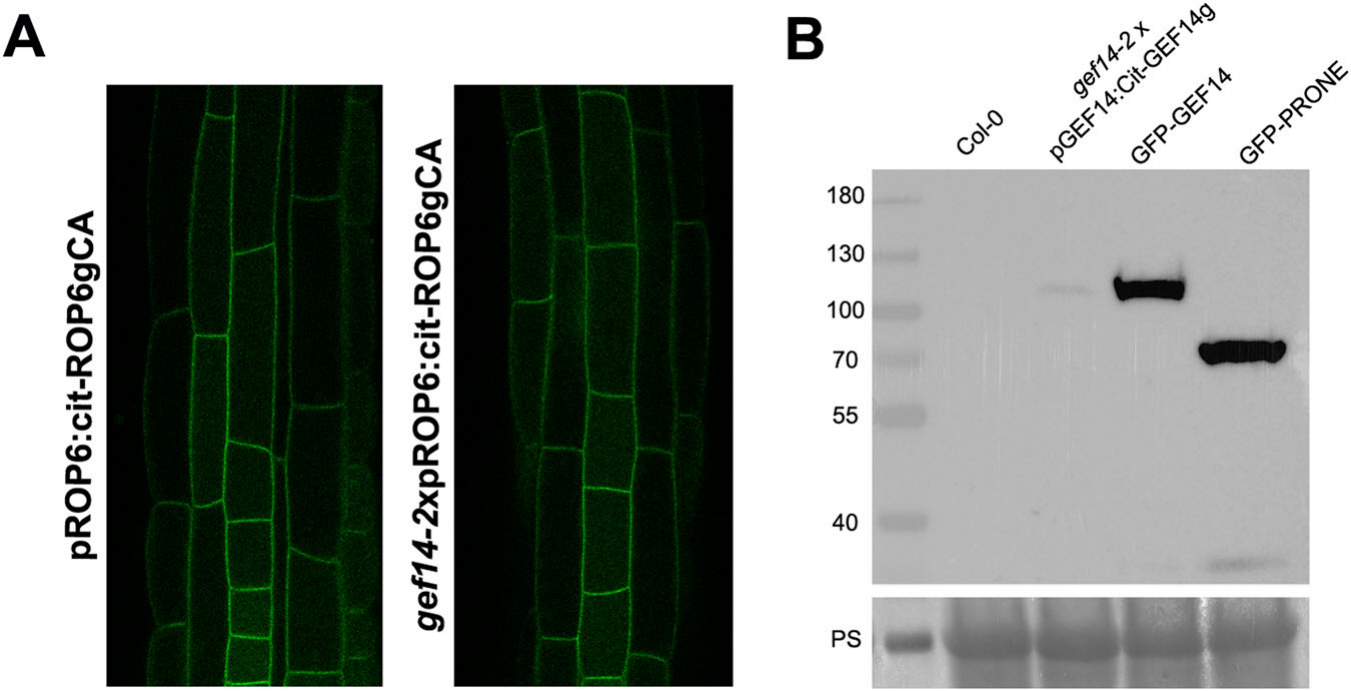
GEF14 do not interfere with ROP6-CA expression pattern and characterization of GEF14 expressing lines. (**A**) Fluorescent signal of pROP6:cit-ROP6gCA and *gef14-2*xpROP6:cit-ROP6gCA in root tip. (**B**) Western blot with an anti-GFP of plant protein extracts from *gef14-2*xpGEF14:Cit-GEF14, GFP-GEF14 and GFP-PRONE. PS, Ponceau red.

Since ROP6 and GEF14 have a partially overlapping expression pattern and they co-cluster after osmotic stimulation, we tested if GEF14 could be an activator of ROP6. First of all, we took advantage of the phenotype induced by pROP6:cit-ROP6gCA expression (Smokvarska et al, 2020). Indeed, the point mutation G15V in ROP6 sequence, locks ROP6 in its GTP bound form (constitutively active, CA). Consequently, the level of ROS present in pROP6:cit-ROP6gCA expressing plants is higher than in wild type ((Smokvarska et al, 2020); Fig. 4A). Interestingly, the level of ROS was comparable between pROP6:cit-ROP6gCA and *gef14-2*xpROP6:cit-ROP6gCA, and their fluorescent signal remained similar (Figs. 4A and EV4A). This suggested that the loss of GEF14 function is not impacting the elevated ROS accumulation triggered by the constitutive active ROP6-CA. This observation is consistent with a hypothesis where GEF14 is a ROP6 activator. To confirm this hypothesis, we developed a FRET-based sensor to record in vivo ROP6 activation state. The design was based on the iROP sensor previously described in Smokvarska et al (2020). Only the fluorescent tags were changed to allow co-expression experiments (Fig. 4B). Firstly, we expressed transiently in tobacco leaf epidermal cells, iROP and iROP-CA that is constitutively in a GTP-bound form (active). As expected, the FRET ratio is higher in iROP6-CA compared to iROP (Fig. 4C,D). This difference in FRET ratio between iROP and iROP-CA is maintained when a membrane bound negative control is co-infiltrated (AtFH1-RFP) (Fig. 4D). When RFP-GEF14 is expressed in cells, a mild change in FRET/Venus was observed (Fig. 4C,D). Because GEF proteins were previously described to be inactive in resting state, we also cloned the PRONE, the GEF14 catalytic domain, in fusion with RFP (Chang et al, 2013). When co-expressed with iROP6, but not iROP-CA, a clear increase of the FRET ratio was observed (Fig. 4C,D). This result showed that the PRONE domain of GEF14 is sufficient to induce ROP6 activation in tobacco leaf cells. We tested if the amount of GEF14 in plant cells could be rate limiting to trigger osmotic signaling. To this purpose, lines overexpressing GFP-GEF14 and GFP-PRONE were generated (Fig. EV4B). Interestingly, neither GFP-GEF14 nor GFP-PRONE showed an enhancement of osmotically-induced ROS accumulation (Fig. 4E). Those results suggest that GEF14 over accumulation is not enough to trigger osmotic signals. Because the PRONE of GEF14 induced iROP activation (Fig. 4D), we wondered why this construct is not generating higher ROS accumulation in Arabidopsis. When observed by TIRF microscopy, the GFP-PRONE signal appeared homogeneously in cells in control conditions and after an osmotic stimulation (Fig. 4F,G). This differs to what was observed with the full-length GEF14 protein (Fig. 4D,F). The lack of GFP-PRONE dynamic recruitment to the cell surface upon osmotic stimulation may explain why overexpression of PRONE is not generating constitutive high ROS accumulation in Arabidopsis root cells.

### GEF14 controls ROP6 dynamic and clustering in response to the osmotic signal

**Figure 5:**
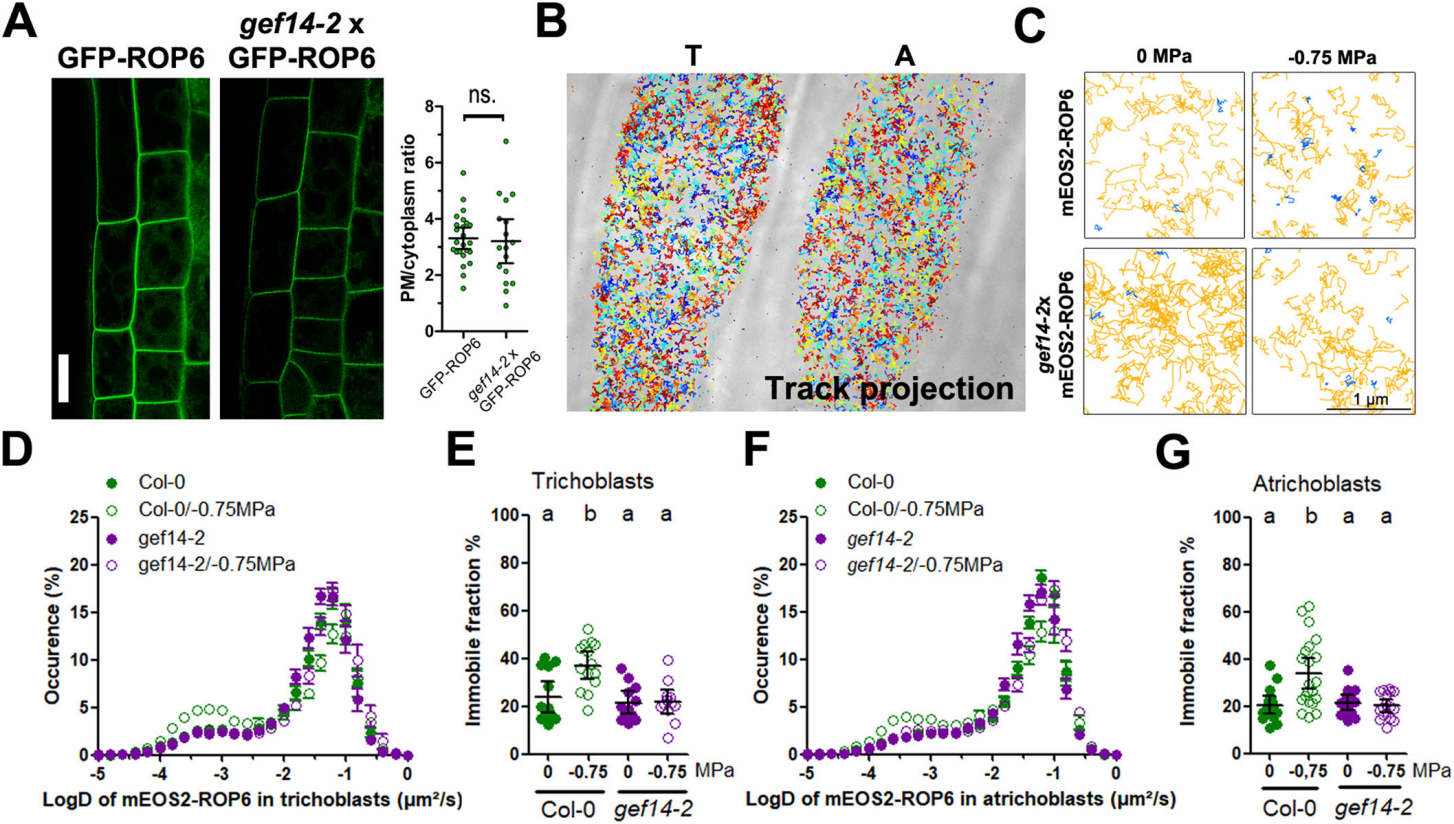
GEF14 modulates ROP6 diffusion and clustering after osmotic stimulation. (**A**) Fluorescent signal from GFP-ROP6 and *gef14-2*xGFP-ROP6 expressing root cells and its representative PM/cytoplasm ratio quantification. (**B**) Image reconstruction from around 6000 single mEOS2-ROP6 molecules in trichoblast (T) or atrichoblast (A) cell. Each color represents a single molecule tracked over time. (**C**) Close-up view of either mEOS2-ROP6 or *gef14-2*xmEOS2-ROP6 expressing trichoblast cells in control or after 15 min treatment with −0.75 MPa solution, where yellow trajectories have a high instantaneous diffusion (upper than 0.01 µm².s^−1^) and blue trajectories a low instantaneous diffusion (below 0.01 µm².s^−1^). (**D**) For trichoblasts (**D**) or atrichoblasts (**F**) bimodal distribution of mEOS2-ROP6 instantaneous diffusion coefficient in Col-0 (green) or *gef14-2* (magenta) in control (close symbol) or after 15 min treatment with −0.75 MPa solution (open symbol). For trichoblasts (**E**) or atrichoblasts (**G**) histogram represents the percentage of mEOS2-ROP6 molecules with an instantaneous diffusion bellow below 0.01 µm².s^−1^ (Log2) in control (close symbol) or after 15 min treatment with −0.75 MPa solution (open symbol) in Col-0 (green) or *gef14-2* (magenta). Mean with Error bars correspond to the 95% confidence interval. According to analysis of variance (ANOVA) followed by a Tukey test, letters indicate significant differences among means (*P* < 0.05). *n* = 17 cells from three independent biological replicas. (**A**) Scale bar 10 µm. a.u., arbitrary units.

We previously showed that ROP6 is forming nanodomains, together with RBOHD/F during cell osmotic stimulation, leading to ROS accumulation (Smokvarska et al, 2020). Our data indicate that GEF14 behave as an activator of ROP6. Therefore, we tested if GEF14 can act on ROP6 localization. At a cellular level, GEF14 has no effect on ROP6 PM binding (Fig. 5A). Thus, we wonder if GEF14 may control ROP6 diffusion and clustering. To test this hypothesis and since the size of ROP6 nanodomain is below the light diffraction limits, we used a single molecule localization microscopy technique (SMLM). Single-particle tracking photoactivated localization microscopy (sptPALM) allows to record diffusion and clustering of several tens of thousands of individual molecules in living cells (Fig. 5B; (Bayle et al, 2021)). As expected, ROP6 diffusion in trichoblast cells is bimodal and osmotic treatment increases the proportion of immobile ROP6 with a D below 0.01 μm^2^/s (Fig. 5C–E). In *gef14-2*xmEOS2-ROP6 trichoblast cells, the osmotic stimulation is not able to reduce mEOS2-ROP6 diffusion (Fig. 5C–E). No impact was found in unstimulated cells (Fig. 5C–E). As GEF14 translational fusion show almost no signal in atrichoblast (Fig. 3B), we wonder if ROP6 is forming osmotically-induced nanodomains in these cells. Interestingly, ROP6 is still able to form nandomains after an osmotic stimulation in atrichoblastic cells, and this occurs in a GEF14-dependent manner (Fig. 5G,H). Those results show that GEF14 is needed to control ROP6 diffusion in response to osmotic stimulus and that atrichoblast cells may behave in a non-autonomous manner for this trait. Alternatively, we cannot exclude that GEF14, although expressed at low levels in atrichoblast cells (i.e., below our detection threshold), is still able to regulate ROP6 diffusion in these cells.

**Figure EV5:**
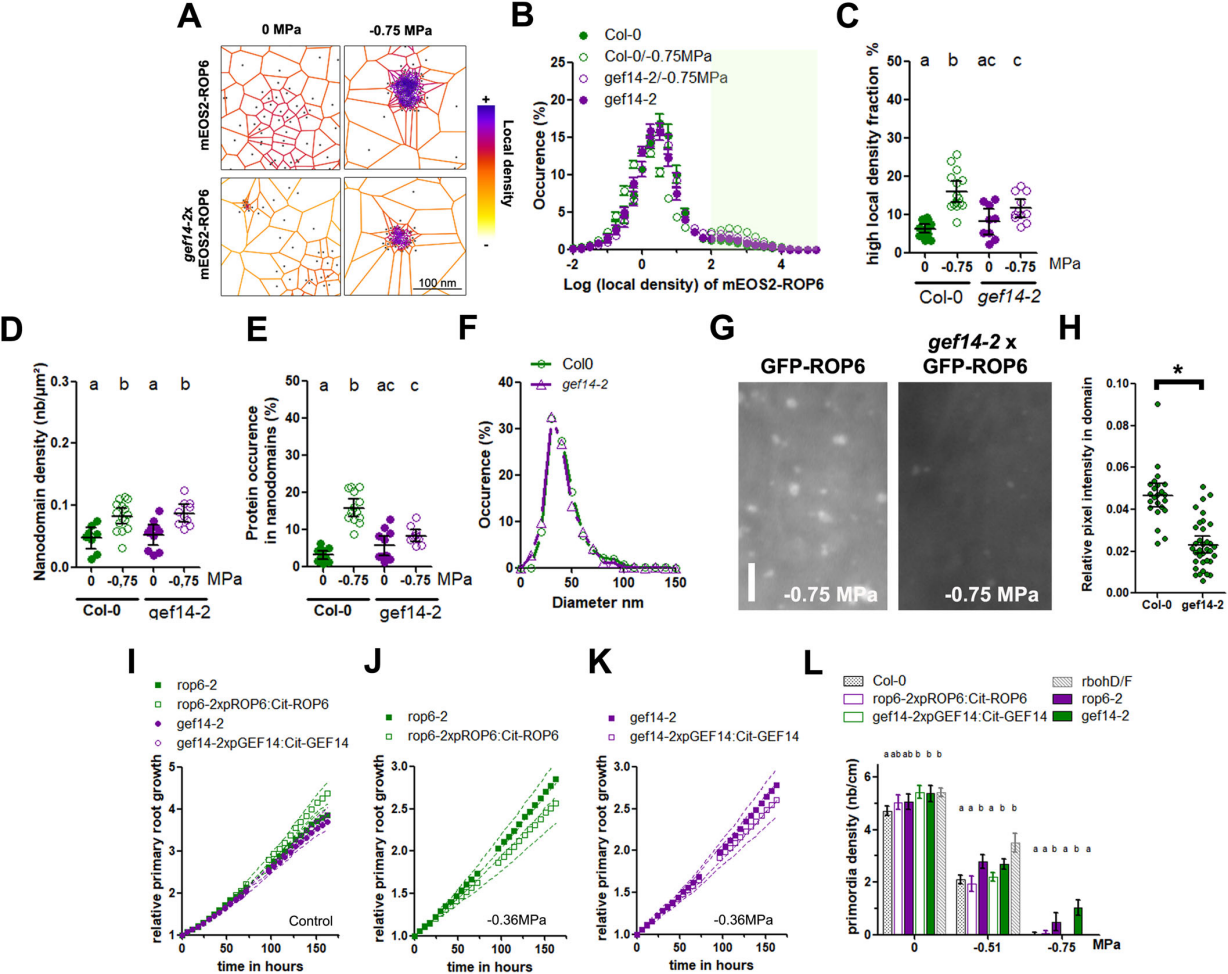
GEF14 interferes with ROP6 abundance in nanodomains and regulates some osmotically triggered downstream responses. (**A**) Voronoï tessellation of mEOS2-ROP6 molecule localization map in control (0 MPa) or after osmotic stimulation (−0.75 MPa) in Col-0 or *gef14-2*. Color code represents the local density of each molecule (labeled as a black circle). (**B**) Distribution of the log of local density of each single molecule in Col-0 (green) or *gef14-2* (magenta) in control (close symbol) or after 15 min treatment with −0.75 MPa solution (open symbol). (**C**) Histogram represents the percentage of mEOS2-ROP6 molecules with a log(local density) higher than 2. (**D**) mEOS2-ROP6 nanodomains density in control (0 MPa) or after 15 min treatment with −0.75 MPa treatment. (**E**) Relative occurrence of mEOS2-ROP6 in nanodomains in control (0 MPa) and treatment (−0.75 MPa) conditions. (**F**) Distribution of the mEOS2-ROP6 nanodomains diameter in control (0 MPa) and treatment (−0.75 MPa) conditions. (**G**) TIRF images of GFP-ROP6 and *gef14-2*xGFP after 15 min treatment with −0.75 MPa treatment. Quantification of ROP6 nanodomains relative GFP signal in ROP6 nanodomains (**H**). (**I**–**K**) Kinetics of the relative primary root growth of *rop6-2*, *gef14-2*, *rop6-2*xpROP6:Cit-ROP6g and *gef14-2*xpGEF14:Cit-GEF14g in control (**I**) or −0.35 MPa plate (**J**, **K**). (**L**) Quantification of primordia density in control, −0.51 MPa or −0.75 MPa plates. Mean with Error bars correspond to the 95% confidence interval. According to analysis of variance (ANOVA) followed by a Tukey test, letters indicate significant differences among means (*P* < 0.05). (**A**–**F**) *n* = 17 cells from three independent biological replicas. (**G**, **H**) *n* > 24 from two independent biological replicas. (**I**–**K**) *n* > 14 plants from two independent biological replicas. (**L**) *n* > 16 plants from two independent replicas. (**H**) *t*-test, *p-value < 0.0001 Scale bar, 10 μm.

Then, we used Voronoï tessellation to calculate ROP6 clustering at the scale of single molecules (Levet et al, 2015). ROP6 local density is induced upon osmotic stimulation (Fig. EV5A–C). Interestingly, this osmotic-dependent increase of the local density is reduce in *gef14-2*x mEOS2-ROP6 (Fig. 5A–C). Indeed, the relative number of ROP6 molecules within ROP6 nanodomains is lower in *gef14-2*xmEOS-ROP6 compare to the control line (Fig. EV5D–F). However, the density and size of the ROP6 PM nanodomains remained unchanged (Fig. EV5D–F). To confirm this role of GEF14 on ROP6 nanodomains formation, we quantified the GFP signal intensity in ROP6 nanodomains seen by TIRF microcopy. After cell osmotic stimulation, we found that the relative pixel intensity is lower in *gef14-2*xGFP-ROP6 trichoblastic cells compare to GFP-ROP6 (Fig. EV5G,H). Together, our results show that GEF14 regulates both ROP6 diffusion and clustering.

To further confirm the role of GEF14 in osmotically-dependent plant responses, we quantify root architecture in response to osmotic signal. *rop6-2* and *gef14-2* show a slightly but not significant faster primary root growth compared to their respective complemented lines (Fig. EV5I,J,K). Recently, it was shown that plant water status could act on lateral root initiation (Mehra et al, 2022). As expected, Col-0 primordia density gradually decrease with the strength of the osmotic stress (Fig. EV5L). This effect is reduced in *rbohd/f*, suggesting a link between ROS signaling and primordia density. Interestingly, whereas no effect on primordia density was observed in control condition, *rop6-2* and *gef14-2* show more primordia compare to Col-0 or complementation lines under osmotic stress (Fig. EV5L). Thus, GEF14 appears to act on ROP6 dynamic that is later needed for osmotically induced ROS and some downstream root developmental parameters like the number of lateral root primordia.

### GEF14 is dispensable for cell responses to auxin signaling

**Figure 6:**
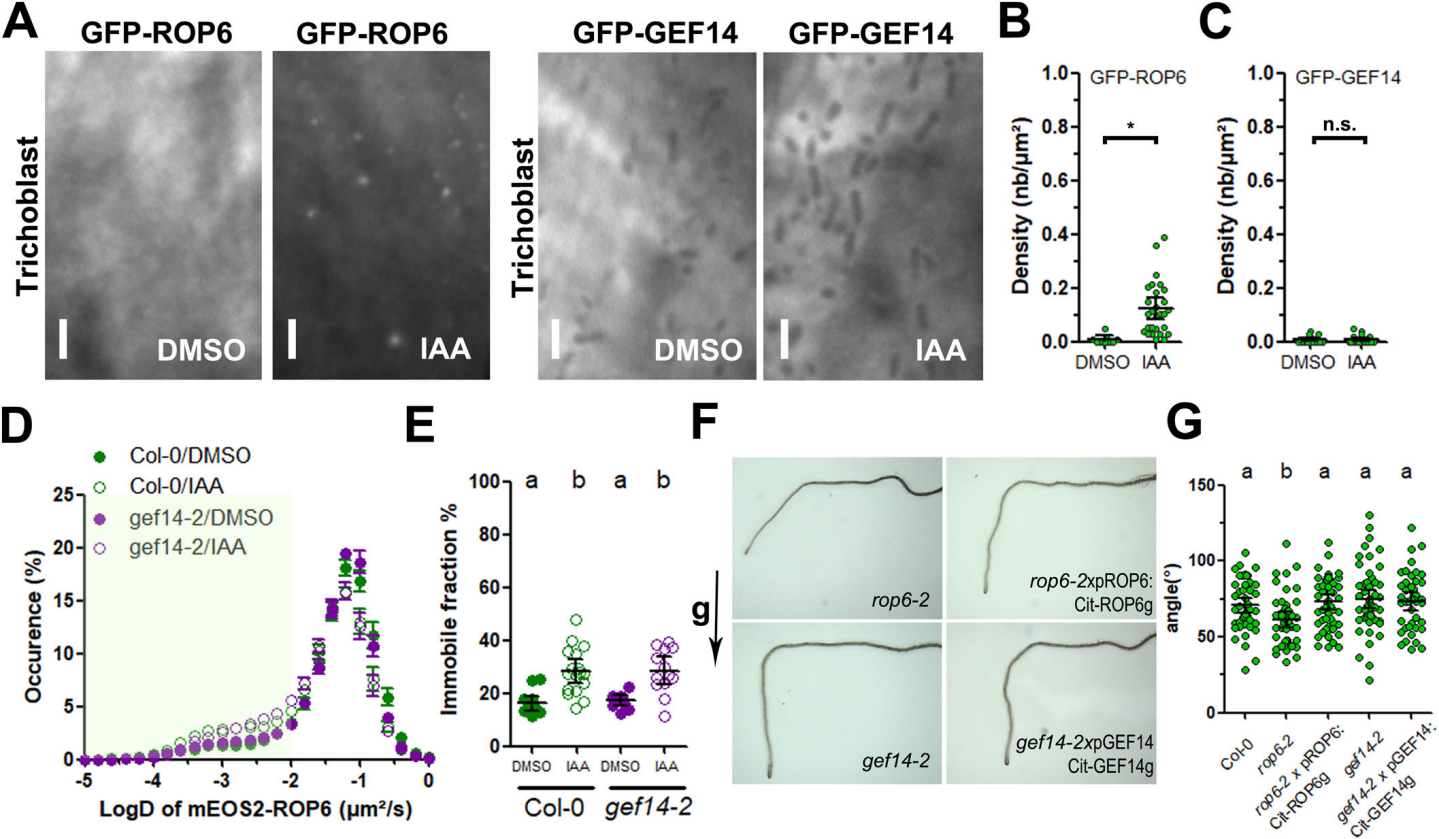
GEF14 is dispensable for cell responses to auxin. (**A**) TIRF images of GFP-ROP6 or GFP-GEF14 expressing trichoblast cells in control condition or after 20 min treatment with 10 µM IAA, and its respective cluster density quantification (**B**, **C**). (**D**) Bimodal distribution of mEOS2-ROP6 instantaneous diffusion coefficient in Col-0 (green) or *gef14-2* (magenta) in control (close symbol) or after 20 min treatment with 10 µM IAA (open symbol). (**E**) Histogram represents the percentage of mEOS2-ROP6 molecules with an instantaneous diffusion bellow below 0.01 µm².s^−1^ in control (close symbol) or after 20 min treatment with 10 µM IAA (open symbol) in Col-0 (green) or *gef14-2* (magenta). (**F**) Picture of the different genotype after gravistimulation. (**G**) Quantification of the root angle 24 h after gravistimulation. Mean with Error bars correspond to the 95% confidence interval. (**B**, **C**) *t*-test, *n* > 20 cells from two independent biological replicas. (**B**) *p-value = 0.0018. (**C**) n.s. non-significant difference, *p*-value = 0.93. (**E**) According to analysis of variance (ANOVA) followed by a Tukey test, letters indicate significant differences among means (*P* < 0.05). *n* > 13 cells from two independent biological replicas. For (**G**), *n* > 31 plants from two independent biological replicas. a.u., arbitrary units.

**Figure EV6:**
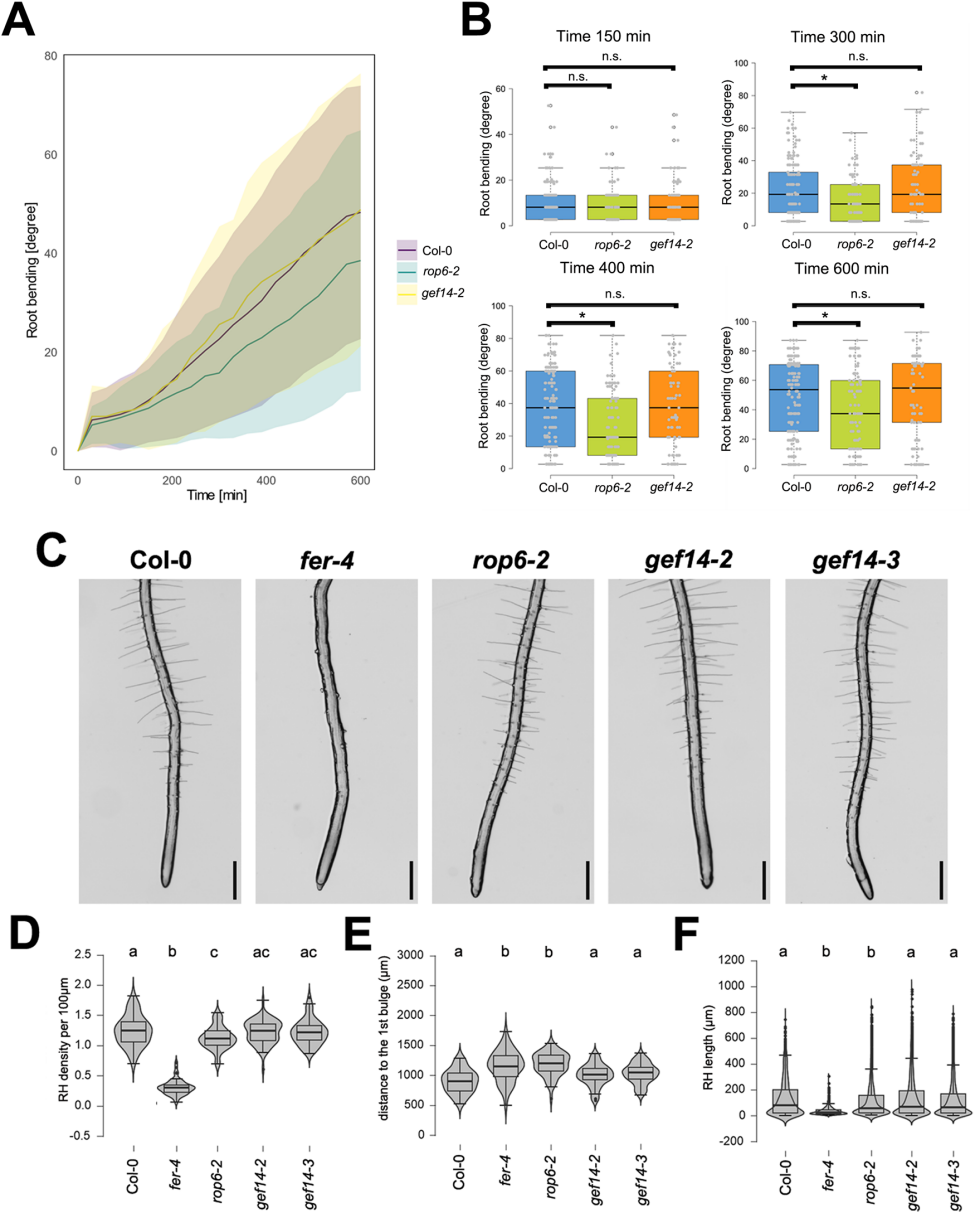
GEF14 is dispensable for root gravitropic response. (**A**) Mean +/− SD root bending (SD represented as shaded areas) angle during 600 min after 90° gravistimulation of Col-0, *rop6-2* and *gef14-2* seedlings. Images were taken every 30 min using spiro. (**B**) Median comparison of root angle after 150, 300, 400, and 600 min. (**C**) Representative images of Col-0, *fer4* (positive control), *rop6-2*, *gef14-2* and gef14-3 root hairs. (**D**–**F**) Quantification of root hair density, distance to the fist bulge and length, respectively. (**B**) Dunnett test, at time 150 min *p*-value (Col0, rop6) = 0.1612 and *p*-value (Col0, ropgef14) = 0.5868, at time 300 min *p*-value (Col0, rop6) = 0.0013 and *p*-value (Col0, ropgef14) = 0.6777, at time 450 min *p*-value (Col0, rop6) = 0.0007 and *p*-value (Col0, ropgef14) = 0.9408 and at time 600 min *p*-value (Col0, rop6) = 0.0089 and *p*-value (Col0, ropgef14) = 0.9331. *n* (Col0, *rop6-2*, *gef14-2*) = 160, 90, 77 roots from 4 independent biological replicas. n.s non-significant, *p-value < 0.05 Dunnett test. (**D**–**F**) Boxplots show median, 1st and 3rd quartile; the whiskers extend to data points <1.5 interquartile range away from the 1st or 3rd quartile; all data points are shown as gray dots. According to analysis of variance (ANOVA) followed by a Tukey test, letters indicate significant differences among means (*P* < 0.05).

Next, we wondered if GEF14 relocalization and its effect on ROP6 diffusion and clustering is also altered after cell stimulation with another signal. Auxin was shown to act on ROP6 diffusion and nanodomain formation that later determine auxin related cellular and tissue responses (Platre et al, 2019; Pan et al, 2020). Firstly, we tested if GEF14 forms nanoclusters after 10 µM IAA application for 20 min, like GFP-ROP6 does (Platre et al, 2019; Smokvarska et al, 2020). TIRF observation show that GFP-ROP6, but not GFP-GEF14 forms nanoclusters at the PM upon auxin treatment (Fig. 6A–C). This result suggests that GEF14 is not responding to auxin application. As previously described, IAA treatment increases the ROP6 immobile fraction, this effect being fully maintained in *gef14-2* line (Fig. 6D,E). Finally, we tested the role of GEF14 in root gravitropic response, been partially controlled by ROP6. As previously described (Lin et al, 2012), *rop6-2* showed a mild agravitropism phenotype complemented in *rop6-2*xpROP6:cit-ROP6g, whereas *gef14-2* behaved like control plant. This effect was shown both during 10 h kinetics and 24 h after gravitropic stimulation of the roots (Figs. 6F,G and EV6A,B). This result suggests that GEF14 is not involved in root gravitropism. Several ROPs and GEFs are known to regulate root hair development (Denninger et al, 2019; Gendre et al, 2019; Hirano et al, 2018). Thus, we quantified root hair density, length and distance to the first bulge of Col-0, *rop6-2* and *gef14-2*. Whereas *rop6-2* showed mild phenotype, *gef14-2* behaved like Col-0 plants (Fig. EV6C–F). Once more, these results suggest that the role of ROP6 on root hair development is not mediated by GEF14. Altogether, those results demonstrated that GEF14 relocalization at the PM and its impact on ROP6 dynamic is restricted to osmotic signal.

## Discussion

By combining genetic, FRET activation sensor and super resolution microscopy, we identified and characterized GEF14 as an osmotic-specific activator of ROP6 signaling pathway. Moreover, we found that GEF14 regulates in a signal-specific manner ROP6 dynamics and nanodomain formation within the PM.

Rho GTPases are classically seen as hub proteins, forming the neck of an hourglass-shaped signaling network. It is especially the case of ROP6 in Arabidopsis root cells. Indeed, in addition to its role in osmotic and auxin signaling, we found that ROP6 is necessary for cell response to ABA and flg22 (Fig. 1, (Platre et al, 2019; Pan et al, 2020)). We also showed that not all ROS-mediated signaling pathways need ROP6. For instance, RALF1 treatment induces ROS accumulation in root cells of wild type plants but also in *rop6-2* (Fig. 1D). RALF1 peptide is known to interact with *Catharanthus roseus* receptor kinases (CrRLKs), especially FERONIA and trigger secondary messengers like ROS, pH variation and calcium influx (Haruta et al, 2014; Malivert and Hamant, 2023). It is possible that RALF1-mediated ROS accumulation is still dependent on RBOHs, like it is for osmotic, ABA or flg22 signaling. But, these responses may require another ROP isoform. Alternatively, they may bypass the requirement for Rho GTPase-dependent activation altogether and instead may rely on kinase-mediated activation like it is for BIK1, SCHENGEN3 or CDPK-dependent RBOHs activation (Drerup et al, 2013; Dubiella et al, 2013; Kadota et al, 2014; Fujita et al, 2020).

We found that ROP6 but not GEF14 is needed to trigger plant response to auxin, ABA and flg22 signals (Fig. 2, (Pan et al, 2023)). Whereas SPK1 is a GEF isoform involved in auxin-dependent ROP6 activation in root cells or how ABA and flg22 activate ROP6 in this tissue is unknown (Qiu et al, 2002; Basu et al, 2008). In stomata, ABA induces SNRK2.4/OST1 that phosphorylate RBOHD and F, leading to ROS accumulation. On the other hand, previous studies revealed that in roots *gef1-1xgef4-1xgef10-1* and *gef1-1xgef4-1xgef10-1xgef14-1* show an ABA hypersensitivity response (Yu et al, 2012; Li et al, 2016, 2018). Under ABA, GEF1, 4, 10, 12, and 14, but not GEF7, form intracellular structures in cells, leading to their degradation (Li et al, 2016). Collectively, these results suggest that GEFs can act as negative regulators of the ABA transduction pathway and potentially downregulate ROS signaling. How this effect can be reconciled with the role of ROP6 on ABA-dependent ROS accumulation in root cells remains at this stage unclear and suggests a complexification of this pathway.

We found that GEF14, known to regulate PVC growth, is needed to mediate some of the plant response to osmotic signals (Figs. 2 and SV5L) (Lin et al, 2021; Tang et al, 2021). In addition of a role on secondary messenger accumulation e.g., ROS, GEF14 acts as negative regulator of lateral root initiation in hyperosmotic condition (Fig. SV5L). This result suggest that ROS could be a negative regulator of primordia initiation during root water stress. This observation is to some extend comparable to root xerobranching response, where local low water potential in soil shaped the root architecture (Mehra et al, 2022, 2023). A role of ROS during xerobranching is discussed in a preprint, which reports that ROS mediates the oligomerization of IAA3/SHY2 (preprint: Roy et al, 2024).

Whereas GEF14 trigger plant response to osmotic stimulation, it is not the case for 6 GEF isoforms that are also expressed in root cells (Fig. EV2). This shows that the osmotic signals induce a pathway that acts specifically on GEF14. Our results obtained in transient expression suggest that full-length GEF14 is weakly active in resting condition (Fig. 4C,D). This is in accordance with the fact that GEF1 was shown to be activated by phosphorylation to regulate ROP1 in pollen tube tip growth (Chang et al, 2013). Therefore, it is possible that a similar post translation regulation happens in the case of osmotic signaling in root cells. The nature of GEF14 activator being a kinase or a phosphatase in the context of osmotic signaling remains unknown at this stage. Nevertheless, in the context of ABA signaling, it was shown that GEF1 is activated by CPK4, and some CPKs were shown to be important to regulate root growth after osmotic related stimulation (Mehlmer et al, 2010; Li et al, 2015, 2018). This gene family might constitute interesting candidates for further investigations.

We found that full-length GEF14, but not its PRONE domain alone, formed stimuli-dependent clusters, that localized within ROP6 nanodomains, suggesting that either GEF14 C or N-ter sequences are important for PM targeting. Whereas the PRONE domain is sufficient to activate ROP6 in heterologous system, we found that its overexpression is not enough to trigger constitutive ROS in Arabidopsis roots, suggesting that plasma membrane association of GEF14 is required for ROP6 activation. Together, those results suggest that the localization of GEF14 in specific domains at the PM, may trigger the recruitment of ROP6 in nanodomains. In a preprint, it was recently discovered that ROP2 activation was not sufficient to induce a proper ROP2 localization at the RHID (preprint: Fuchs et al, 2021). This suggest that in addition to its role in ROP activation, GEF localization is of importance to convey information for proper signal transduction in cells.

GEF14 is able to control ROP6 dynamics and nanodomains formation in response to an osmotic signal (Figs. 5E,H and SV5C,E). This effect seems to be true for both trichoblasts and trichoblasts cells, while GEF14 was not detectable in this last cell type (Figs. 5 and 3). Even if this need further confirmations, this observation suggest that atrichoblast cells can react to osmotic stimuli in non-cell autonomous manner. Interestingly, the role of GEF14 is restricted to the osmotic signal since we showed that GEF14 is dispensable for auxin-mediated ROP6 nanodomains formation, the root gravitropic response and root hair development (Figs. 6E,G and SV6D–F). Thus, GEFs can control signal specific ROP6 nanodomains formation. Because, the composition of ROP6 nanodomains vary depending of the upstream signal, we can therefore conclude that GEFs participate in the establishment of signal specificity in Arabidopsis root cells.

## Methods

**Table:** Reagents and tools table

Reagent/Resource	Reference or Source	Identifier or Catalog Number
**Experimental models**		
*rop6-2*	Lin et al, 2012	SALK8091737C
*gef2-1*	Wang et al, 2017	Salk_130229
*gef3-1*	Denninger et al, 2019	Salk_079879C
*gef4-2*	Denninger et al, 2019	SALK_107520
*gef10-1*	Denninger et al, 2019	Salk_009456C
*gef11-1*	Denninger et al, 2019	SALK_126725
*gef14-2*	Denninger et al, 2019	Salk_046067
*gef14-3*	Li et al, 2016	SAILseq_444_H11
*gef1/4/10*	Li et al, 2016	NASC ID (N69175) gef1 (SALK_058164C), gef4 (CS808780), gef10(SALK_009456C).
*gef1/4/10/14*	Li et al, 2016	NASC ID (N69176) gef1(SALK_058164C), gef4 (CS808780), gef10(SALK_009456C), gef14 (CS820474).
*rop6-2xpROP6:cit-ROP6*	Platre et al, 2019	
*rop6-2xpROP6:cit-ROP6-CA*	Platre et al, 2019	
*P35s:GFP-ROP6*	Smokvarska et al, 2020	
*PUBQ:mEOS2-ROP6*	Platre et al, 2019	
*gef3-1xpGEF14:cit-GEF14*	In this study	
*gef14-2xpGEF14:cit-GEF14*	In this study	
*gef14-3xpROP6:cit-ROP6-CA*	In this study	
*gef14-2xP35s:GFP-ROP6*	In this study	
*gef14-2xPUBQ:mEOS2-ROP6*	In this study	
*P35s:GFP-GEF14*	In this study	
*P35s:GFP-PRONE*	In this study	
**Recombinant DNA**		
*pGEF14:cit-GEF14*	Denninger et al, 2019	
*P35s:AtFH1-RFP*	Martinière et al, 2011	
*P35s:GFP-GEF14*	In this study	
*P35s:GFP-PRONE*	In this study	
*P35s:RFP-GEF14*	In this study	
*P35s:RFP-PRONE*	In this study	
p35s:PAK1-mTFP-mVenus-ROP6g-CA	In this study	
p35s:PAK1-mTFP-mVenus-ROP6g	In this study	
**Antibodies**		
Anti-GFP	Milteny	clone GG4-2C2.12.10
**Chemicals, Enzymes and other reagents**		
NaCl	Sigma-Aldrich	57653-1 kg
NaOH	Sigma-Aldrich	55881-1 kg
TG-SDS 10x	Euromedex	EU0510-B
Tris	Sigma-Aldrich	252859-500 g
HCL 37%	VwR-PROLABC	20252.290 1 L
SDS	Euromedex	EU0660-A
DTT	Sigma-Aldrich	43817 5 g
Tween 20	Sigma-Aldrich	P7949 100 ml
Murashige and Skoog	Sigma-Aldrich	M5519 1 L
Sucrose	Sigma-Aldrich	S5390 1 kg
MES	Euromedex	EU0033
KOH	Sigma-Aldrich	P5958 1 kg
DHE	Sigma-Aldrich	07008 10 mg
IAA	Sigma-Aldrich	
ABA	Sigma-Aldrich	41049 250 mg
DMSO	Sigma-Aldrich	D4540 100 ml
Sorbitol	Sigma-Aldrich	S1876 1 kg
Flg22	Proteogenix	183294
RALF1	Proteogenix	ATTKYISYQSLKRNSVPCSRRGASYYNCQNGAQANPYSRGCSKIARCRS (95.2%)
**Software**		
Fiji	https://imagej.net/software/fiji	
Trackmate	https://imagej.net/plugins/trackmate/	
sptPALM_viewer	Bayle et al, 2021	
SR-Tesseler	Levet et al, 2015	
Ilastik	https://www.ilastik.org/	
RootSystemTracker	Fernandez et al, 2022	
Gravitropic index	SiCE Macro gravi	https://github.com/RDP-vbayle/SiCE_FIJI_Macro/tree/main/RootGravi
Root hair analysis		https://github.com/RDP-vbayle/SiCE_FIJI_Macro/blob/main/misc/ MacroRootHair%20240626batchZoom3.ijm

### Growing condition and plant material

Arabidopsis thaliana accession Col-0 (N60000) was used as wild type in this study. The following lines were published before: *rop6-2*, *gef2-1*, *gef3-1*, *gef4-2*, *gef10-1*, *gef11-1*, *gef14-2*, *gef14-3*, *gef1/4/10*, *gef1/4/10/14*, rop6-2xpROP6-cit-ROP6g, rop6-2xpROP6-cit-ROP6g-CA, GFP-ROP6, mEOS2-ROP6 (Li et al, 2016; Denninger et al, 2019; Platre et al, 2019; Smokvarska et al, 2020). Plants were stratified for 2 days at 4 °C and grown vertically on agar plates containing half-strength Murashige and Skoog (MS) medium supplemented with 1% (w/v) sucrose, 2.5 mM MES-KOH pH 5.7, 0.8% Agar typeE for 5 days at 22 °C in a 16-h light/8-h dark cycle with 70% relative humidity and a light intensity of 200 μmol·m^−2^·s^−1^, prior to use. *Nicotiana tabacum* (SR1) used for transient expression were grown in soil at 22 °C in a 8-h light/16-h dark cycle with 70% relative humidity and a light intensity of 200 μmol·m^−2^·s^−1^.

### Cloning and plant transformation

*GEF14* and *GEF14* PRONE (from amino acids residues 127 to 500) sequence was PCR amplified from seedling cDNA and cloned in pENTR/DTOPO. pB7WGF2 and pB7WGR2 vector were used as destination vector for respectively GFP and RFP fusion. pGEF14:cit-GE14 was obtained from Denninger et al, (2019). The unimolecular FRET sensor with intact C terminus was designed based on iROP biosensors (Smokvarska et al, 2021). The CRIB domain of hsPAK1 is known to interact with GTP bound form of ROP (Tao et al, 2002; Akamatsu et al, 2013). We used it as a genetic probe for ROP6 GTP conformation. We ordered a synthetic gene coding for PAK1-mTFP-mVenus (iROP) and cloned it into *pDONR221*. Tripartite gateway cloning strategy were used to generate p35s:PAK1-mTFP-mVenus-ROP6g or p35s:PAK1-mTFP-mVenus-ROP6g-CA. The different binary were used either for transient expression in tobacco or to generate stable *Arabidopsis* plants by floral dip method in Col-0 or *gef14-2*. The following crosses were made in this study: *gef14-2*xGFP-ROP6, *gef14-2*xmEOS2-ROP6, *gef14-2*xpROP14-cit-GEF14, *gef14-2*xpROP6-cit-ROP6g-CA and *gef3-1*xpGEF14:cit-GEF14.

### ROS measurement

Five-day-old plantlets were incubated in a liquid resting buffer (half-strength Murashige and Skoog (MS) medium supplemented with 1% (w/v) sucrose, 2.5 mM MES-KOH pH6 for 30 min to allow recovery from transfer. Then, plantlets were gently transferred for an additional 15 min with 5 μM of ROS dye dehydroethidium (DHE) into resting buffer (0 MPa) or resting buffer with 300 mM sorbitol (−0.75 MPa). When indicated, DHE co-treatment with flg22 (1 μM, 30 min), RALF1 (1 µM, 15 min) or ABA (1 μM, 1 h) was applied. Observations were performed on the root elongation zone using an Axiovert 200 M inverted fluorescent microscope (20×/0.5 objective; Zeiss), with 512/25-nm excitation and 600/50 emission filters. Exposure time was 500 ms. Images were acquired using a CCD camera (Cooled SNAP HQ; PhotoMetrics), controlled by imaging software (MetaFluor; Molecular Devices). To quantify the intensity of the fluorescence signal, the images were analyzed using ImageJ software. After subtraction of the background noise, an average mean gray value was calculated only from the root epidermal cells.

### Western blot

Tissues from 5-day-old plantlets were grinded with liquid nitrogen to a fine powder and resuspended in 300 µl/100 mg powder of extraction buffer (125 mM Tris-HCl, pH 6.8, 2.5% SDS, 20% glycerol, 0.01% bromophenol blue and 4% DTT). Western blot analysis was performed with HRP GFP antibodies (Milteny, clone GG4-2C2.12.10) at 1:10,000 in blocking solution (2% skim milk in 0.1% Tween-20 and TBS).

### Confocal and FRET measurement

Plant samples were imaged using Leica SP8 microscope with a 40×/1.1 water objective. 488-nm excitation and 500–540 nm emission was used for GFP detection. 512-nm excitation and 520–540 nm emission was used for mCitrine (cit) detection. For FRET measurement, images were taken with 458-nm excitation wavelenght and a simultaneous detection between 464–500 nm (mVenus detection) and 514–550 nm (FRET channel). The ratio of FRET/Venus images was calculated with a Fiji software. Mean gray value of each cells present in the field of view was measured by drawing ROI.

### TIRF microscopy

For cluster density analysis, Total Internal Reflection Fluorescence (TIRF) microscopy was performed on a custom-made microscope using an inverted Zeiss microscope and a 100x/1.46 oil immersion objective (Apochromat NA = 1.46; Zeiss). An emCCD camera (iXON XU_897; ANDOR) was used for image acquisition. TIRF illumination was allowed using 405 nm or 488 nm laser excitations (LBX 405 and 488 50 mW; OXXIUS). Dichroic and emission filters (Chroma) were selected according to GFP and mVenus fluorescence spectra.

Trichoblasts cell files were first manually identified from root hair bulge in the differentiation zone using brightfield illumination. Then, cell files were followed until reaching the root elongation zone. TIRF acquisition was recorded for 5 s (100 frames), with 50 ms exposure time and a gain set at 300. The 100 images were then averaged and object detection was performed using machine learning segmentation with Ilastik (Levet et al, 2015). Segmented images were imported in Fiji software to determine the number of clusters in a ROI of 100 × 100 px with pixel size of 0.102 nm.

### sptPALM

Root cells were observed with a homemade total internal reflection fluorescence microscope equipped with an electron-multiplying charge-coupled device camera (Andor iXON XU_897) and a 100×/1.46 oil immersion objective. The coverslips (Marienfeld 1.5H) were washed sequentially with 100% ethanol, acetone and water. Then, they were bathed with a 1 M KOH solution and then ultra-sonicated for 30 min. After several wash-outs with MilliQ water, they were dried under Bunsen burner flame. The laser angle was adjusted so that the generation of the evanescence waves give a maximum signal-to-noise ratio. The activation of the photoconvertible tagged mEOS-ROP6 and gef14-2xmEOS2-ROP6 was done by a low-intensity illumination at 405 nm (LBX 405 50 mW; OXXIUS), and 561 nm (SAPPHIRE 100 mW; Coherent) emission combined with a 600/50 (Semroch) emission filter was used for image acquisition. Seven-thousand images were recorded per region of interest at 30 ms exposure time. Five to seven trichoblast cells/treatment were analyzed out of two to three biological replicates.

### Single-particle tracking and voronoï tesselation

Individual single molecules were localized and tracked using the trackmate software (Tinevez et al, 2017). Dynamic properties of single emitters in root cells were then inferred from the tracks using a homemade analysis software, palm_viewer written in MATLAB (The MathWorks) (Bayle et al, 2021). From each track, the MSD was computed. To reduce the statistical noise while keeping a sufficiently high number of trajectories per cell, tracks of at least seven steps were used. Missing frames due to mEOS2 blinking were allowed up to a maximum of three consecutive frames. The instantaneous diffusion coefficient (D) was then calculated by fitting the MSD curve using the first four points. Voronoï tessellation was calculated from the localization of single ROP6 molecules extracted from TrackMate. Correction for multiple detection of single emitters was made on the basis of recommendation from Levet et al, 2015. The local density of each molecules were calculated as the invert of their minimal polygonal surface. Then, nanocluster size, relative number of ROP6 molecules in nanodomaines, and density of nanodomains were calculated after defining ROI where the local density was 50 times higher than the average. Only ROIs with at least 25 detections were considered.

### Primary root growth

The different genotypes were grown for 5 days in Hoagland media at 23 °C, 65% humidity, 16 h days and 150 µE. Then, plantlets were transfer on either control plate (Hoaglande media) or plate supplemented with 0.150 mM sorbitol (−0.36 MPa). Image acquisition and analysis were performed as described previously (Fernandez et al, 2022). In brief, we utilized the HIgh Resolution ROot Scanner (HIRROS) setup for automated and non-destructive visualization of root growth and architecture in *Arabidopsis thaliana*. Images were captured during 6 days and half with an acquisition every 6 h with a resolution of 19 μm/px. The images were then analyzed using the automatic analysis pipeline, RootSystemTracker. The reconstructed architecture is provided in Root System Markup Language (RSML) format (Lobet et al, 2015). RSML files are subsequently processed by automatic routines to extract traits of interest.

### Root primordia density

The different genotypes were grown for 5 days and then transfer to control, 200 mM (−0.51 MPa) or 300 mM sorbitol (−0.75 MPa) plates. After 6 days, the newly grown roots were measured and fixed in 20%EtOH. Number of root primordia were counted with an Olympus BH2 microscope equipped with a 20x dry objective.

### Gravitropic stimulation assays

Three-day-old seedlings were placed in the dark. 24 h later, the seedlings were transferred to new agar plates and kept in the dark. After 1 h, the plates were rotated 90° anticlockwise. 24 h later, roots were scanned and root angles were measured by ImageJ software (NIH; http:// rsb.info.nih.gov/ij). For the spiro-based gravitropic assay (Ohlsson et al, 2024), plants were stratified for 2 days at 4 °C and grown vertically on agar plates containing half-strength MS medium supplemented with 2.5 mM MES (pH 5.7) and 0.8% agar for 5 days at 22 °C in a 16-h light/8-h dark cycle with 70% relative humidity and a light intensity of 200 μmol·m^−2^·s^−1^, prior to use. Seedlings were then transferred in vertical to plates containing the same media and let to recover in a growth chamber. After an hour, the plates were installed in the spiro in the same growth chamber, turned 90° and imaged every 30 min. For the analysis, stacks of images were cropped per genotype and the home-made FIJI script “SiCE Macro gravi” used (https://github.com/RDP-vbayle/SiCE_FIJI_Macro/tree/main/RootGravi).

### Root hair density, length, and distance to the first bulge

For root hair (RH) measurements, 6-day-old seedlings were imaged at zoom 3x using a manual stereomicroscope Nikon SMZ18 equipped with a LED diascopic illumination base and a Camera Hamamatsu Orca Flash4.0LT. Measurements were done using a custom FIJI macro script available at https://github.com/RDP-vbayle/SiCE_FIJI_Macro/blob/main/misc/ MacroRootHair%20240626batchZoom3.ijm. Briefly main root is segmented using Variance filtering followed by distance map thresholding. Root tip is deduced as the lower extremity of skeletonized version of main root. RH segmentation is done using a Top hat filter followed by Classical Thresholding. Main root is subtracted to resulting thresholded object, RH are then converted to ROIs using Analysed Particle. Each root hair ROI is skeletonized, allowing branched RH to be removed as resulting of multiple RH segmentation. Mean Signal and Standard Deviation measurements are used to discriminate between in and out of focus RH.

### Statistical analysis

For each condition or treatment, 9–12 cells were analyzed from at least 5–7 different seedlings. No blinding was done. All experiments were independently repeated 2–3 times. Data are expressed as mean ± 95% confidence interval. ANOVA followed by Tukey test was done, letters indicate significant differences among means (*p*-value < 0.01). ∗*p* value below 0.05 Student t-test. Statistical analyses were performed in GraphPad Prism (GraphPad Software).

## Supplementary information

Peer Review File

Expanded View Figures

## Data Availability

Source data for the figures are available at 10.57745/BEN34J. The source data of this paper are collected in the following database record: biostudies:S-SCDT-10_1038-S44319-025-00412-w.
