# Supplementary material for: GEF14 acts as a specific activator of the plant osmotic signaling pathway by controlling ROP6 nanodomain formation
Source: EMBO Rep. 2025 Mar 13;26(8):2146–65. doi: 10.1038/s44319-025-00412-w (PMC12019552; doi:10.1038/s44319-025-00412-w)
Supplement: Supplementary file 2 — Expanded View Figures [file 44319_2025_412_MOESM2_ESM.pdf]

Expanded View Figures

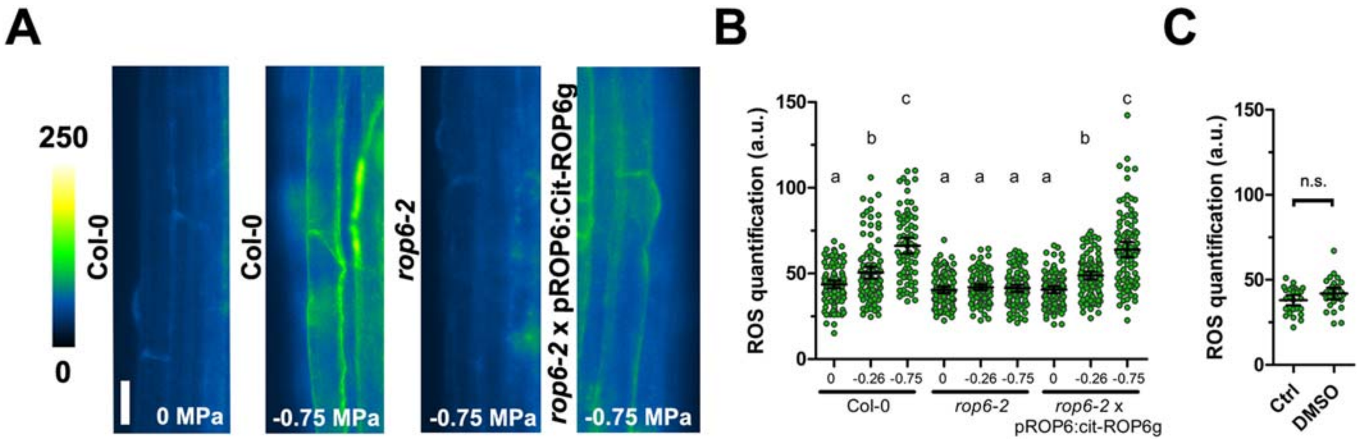

Figure EV1. ROP6 is needed for osmotically-induced ROS accumulation.

(**A**) Images of dihydroethidium (DHE) stained root cells of Col-0, *rop6-2* or *rop6-2*xpROP6:cit-ROP6g in control condition (0 MPa) or after application −0.75 MPa solution. (**B**) DHE fluorescence quantification in control condition, after 15 min treatment with −0.75 MPa solution with the different genetic material. (**C**) DHE fluorescence quantification in control condition or with DMSO. Mean with Error bars correspond to the 95% confidence interval. (**B**) According to analysis of variance (ANOVA) followed by a Tukey test, letters indicate significant differences among means ($P < 0.05$). (**C**) *t*-test, n.s. non-significant difference, *p*-value = 0.089. $n > 31$ cells from three to four independent biological replicas. Scale bar, 10 µm. a.u., arbitrary units.

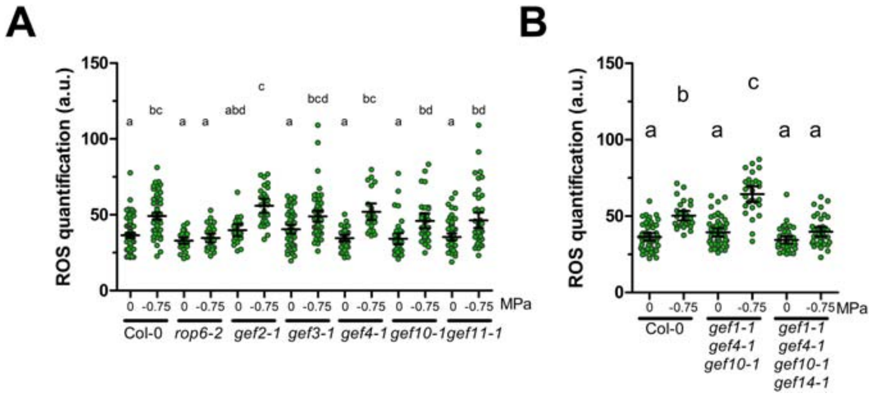

Figure EV2. Characterization of osmotically-induced ROS accumulation in various *GEFs* knock-out lines.

(**A**) DHE fluorescence quantification in control condition or after 15 min treatment with −0.75 MPa solution on different single *GEF* knock-out lines or triple and quadruple GEF mutants lines. (**B**) Mean with Error bars correspond to the 95% confidence interval. (**B**) According to analysis of variance (ANOVA) followed by a Tukey test, letters indicate significant differences among means ($P < 0.05$). $n > 21$ cells from three independent biological replicas. a.u. arbitrary units.

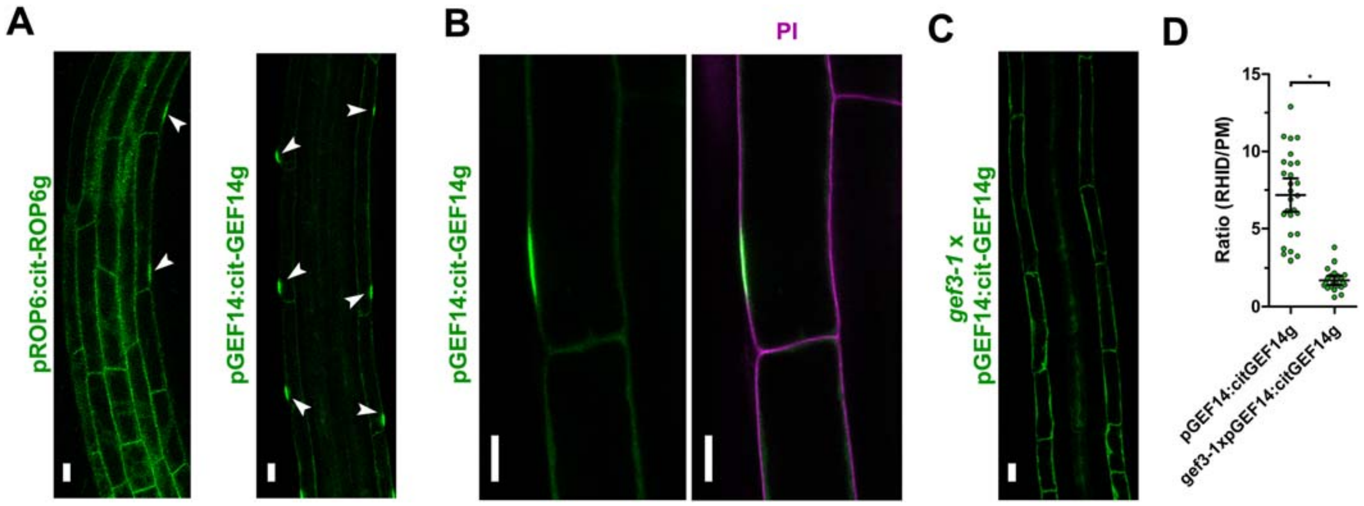

Figure EV3. GEF14 RHID localization is controlled by GEF3.

(**A**) Fluorescent signal of rop6-2xpROP6:cit-ROP6g and *gef14-2*xpGEF14:cit-GEF14g in root tip (arrows show RHID). (**B**) Close-up view of *gef14-2*xpGEF14:cit-GEF14g signal with a propidium iodide (PI) counter staining. (**C**) Fluorescent signal of *gef3-1*xpGEF14:cit-GEF14g in root tip and its respective ratio (RHID/cytoplasm) quantification (**D**). Mean with Error bars correspond to the 95% confidence interval (**D**) *t*-test, *p*-value < 0.0001. *N* > 27 cells from two independent experiments. White arrow point to the root hair initiation domain (RHID). Scale bar, 10 µm.

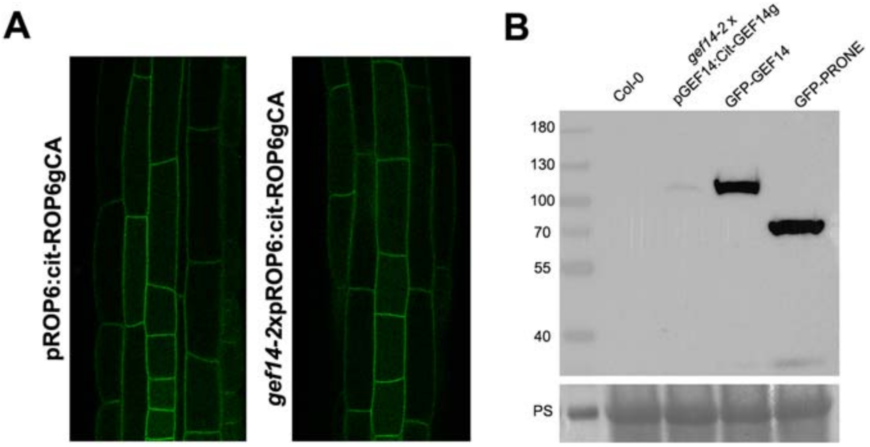

Figure EV4. GEF14 do not interfere with ROP6-CA expression pattern and characterization of GEF14 expressing lines.

(**A**) Fluorescent signal of pROP6:cit-ROP6gCA and *gef14-2*xpROP6:cit-ROP6gCA in root tip. (**B**) Western blot with an anti-GFP of plant protein extracts from *gef14-2*xpGEF14:Cit-GEF14, GFP-GEF14 and GFP-PRONE. PS, Ponceau red.

 © The Author(s)

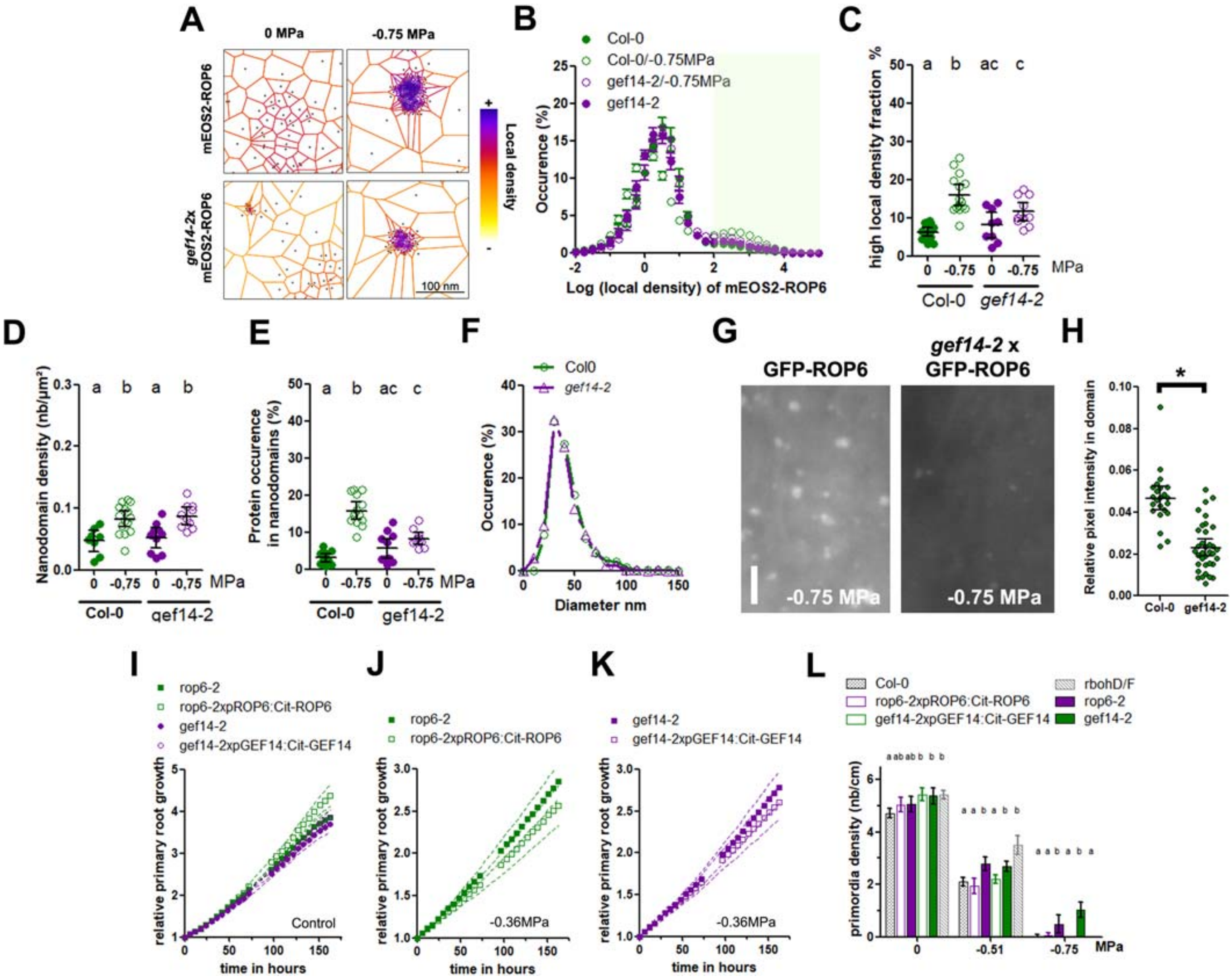

Figure EV5. GEF14 interferes with ROP6 abundance in nanodomains and regulates some osmotically triggered downstream responses.

(**A**) Voronoï tessellation of mEOS2-ROP6 molecule localization map in control (0 MPa) or after osmotic stimulation (−0.75 MPa) in Col-0 or *gef14-2*. Color code represents the local density of each molecule (labeled as a black circle). (**B**) Distribution of the log of local density of each single molecule in Col-0 (green) or *gef14-2* (magenta) in control (close symbol) or after 15 min treatment with −0.75 MPa solution (open symbol). (**C**) Histogram represents the percentage of mEOS2-ROP6 molecules with a log(local density) higher than 2. (**D**) mEOS2-ROP6 nanodomains density in control (0 MPa) or after 15 min treatment with −0.75 MPa treatment. (**E**) Relative occurrence of mEOS2-ROP6 in nanodomains in control (0 MPa) and treatment (−0.75 MPa) conditions. (**F**) Distribution of the mEOS2-ROP6 nanodomains diameter in control (0 MPa) and treatment (−0.75 MPa) conditions. (**G**) TIRF images of GFP-ROP6 and *gef14-2*xGFP after 15 min treatment with −0.75 MPa treatment. Quantification of ROP6 nanodomains relative GFP signal in ROP6 nanodomains (**H**). (**I–K**) Kinetics of the relative primary root growth of *rop6-2*, *gef14-2*, *rop6-2*xpROP6:Cit-ROP6g and *gef14-2*xpGEF14:Cit-GEF14g in control (**I**) or −0.35 MPa plate (**J, K**). (**L**) Quantification of primordia density in control, −0.51 MPa or −0.75 MPa plates. Mean with Error bars correspond to the 95% confidence interval. According to analysis of variance (ANOVA) followed by a Tukey test, letters indicate significant differences among means ($P < 0.05$). (**A–F**) $n = 17$ cells from three independent biological replicas. (**G, H**) $n > 24$ from two independent biological replicas. (**I–K**) $n > 14$ plants from two independent biological replicas. (**L**) $n > 16$ plants from two independent replicas. (**H**) *t*-test, **p*-value < 0.0001 Scale bar, 10 µm.

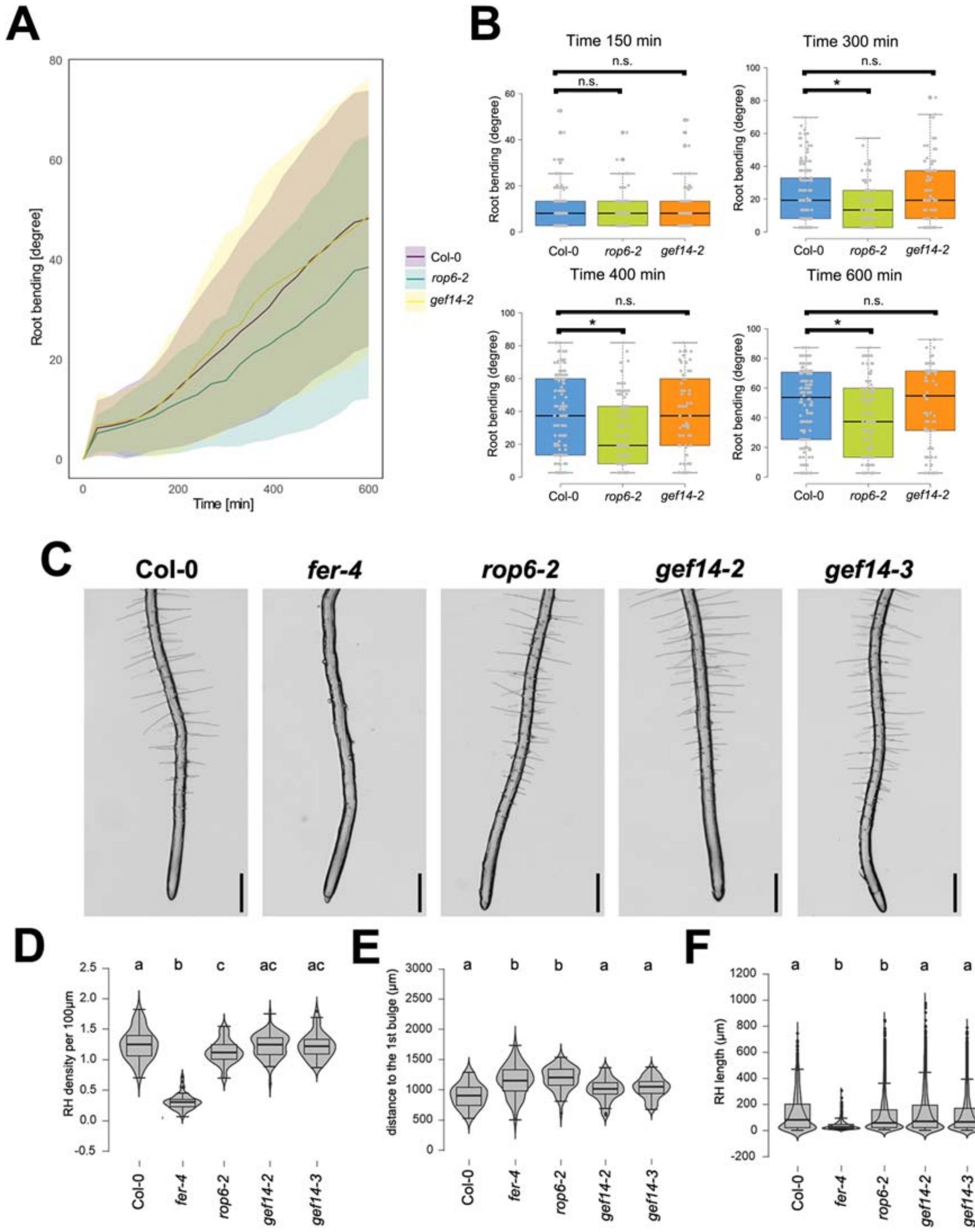

Figure EV6. GEF14 is dispensable for root gravitropic response.

(**A**) Mean +/− SD root bending (SD represented as shaded areas) angle during 600 min after 90° gravistimulation of Col-0, *rop6-2* and *gef14-2* seedlings. Images were taken every 30 min using spiro. (**B**) Median comparison of root angle after 150, 300, 400, and 600 min. (**C**) Representative images of Col-0, *fer4* (positive control), *rop6-2*, *gef14-2* and gef14-3 root hairs. (**D–F**) Quantification of root hair density, distance to the fist bulge and length, respectively. (**B**) Dunnett test, at time 150 min *p*-value (Col0, rop6) = 0.1612 and *p*-value (Col0, ropgef14) = 0.5868, at time 300 min *p*-value (Col0, rop6) = 0.0013 and *p*-value (Col0, ropgef14) = 0.6777, at time 450 min *p*-value (Col0, rop6) = 0.0007 and *p*-value (Col0, ropgef14) = 0.9408 and at time 600 min *p*-value (Col0, rop6) = 0.0089 and *p*-value (Col0, ropgef14) = 0.9331. *n* (Col0, *rop6-2*, *gef14-2*) = 160, 90, 77 roots from 4 independent biological replicas. n.s non-significant, **p*-value < 0.05 Dunnett test. (**D–F**) Boxplots show median, 1st and 3rd quartile; the whiskers extend to data points <1.5 interquartile range away from the 1st or 3rd quartile; all data points are shown as gray dots. According to analysis of variance (ANOVA) followed by a Tukey test, letters indicate significant differences among means ($P < 0.05$).

 © The Author(s)
