# Supplementary material for: GEF14 acts as a specific activator of the plant osmotic signaling pathway by controlling ROP6 nanodomain formation
Source: EMBO Rep. 2025 Mar 13;26(8):2146–65. doi: 10.1038/s44319-025-00412-w (PMC12019552; doi:10.1038/s44319-025-00412-w)
Supplement: Supplementary file 1 — Peer Review File [file 44319_2025_412_MOESM1_ESM.pdf]

EMBO reports

GEF14 acts as a specific activator of plant osmotic signaling pathway by controlling ROP6 nanodomain

Lucille Gorgues, Marija Smokvarska, Caroline Mercier, Clara Igisch, Amandine Crabos, Armelle Dongois, Vincent Bayle, Jean Bernard Fiche, Philippe Nacry, Marcelo Nollmann, Yvon Jaillais, and Alexandre Martinière

Corresponding author(s): Alexandre Martinière (alexandre.martiniere@cnrs.fr)

Review Timeline:

Submission Date:	6th Aug 24
Editorial Decision:	12th Sep 24
Revision Received:	9th Jan 25
Editorial Decision:	29th Jan 25
Revision Received:	10th Feb 25
Accepted:	18th Feb 25

Editor: Martina Rembold

Transaction Report:

(Note: With the exception of the correction of typographical or spelling errors that could be a source of ambiguity, letters and reports are not edited. Depending on transfer agreements, referee reports obtained elsewhere may or may not be included in this compilation. Referee reports are anonymous unless the Referee chooses to sign their reports.)

Dear Dr. Martinière

Thank you for the submission of your research manuscript to our journal. We have now received the full set of referee reports that is copied below.

As you will see, the referees acknowledge that the findings are interesting but they also raise a number of concerns and have suggestions how to further strengthen the data, which need to be addressed.

Given these constructive comments, we would like to invite you to revise your manuscript with the understanding that the referee concerns (as detailed above and in their reports) must be fully addressed and their suggestions taken on board. Please address all referee concerns in a complete point-by-point response. Acceptance of the manuscript will depend on a positive outcome of a second round of review. It is EMBO Reports policy to allow a single round of revision only and acceptance or rejection of the manuscript will therefore depend on the completeness of your responses included in the next, final version of the manuscript.

We realize that it is difficult to revise to a specific deadline. In the interest of protecting the conceptual advance provided by the work, we recommend a revision within 3 months (December 13th). Please discuss the revision progress ahead of this time with the editor if you require more time to complete the revisions.

I am also happy to discuss the revision further via e-mail or a video call, if you wish.

*****IMPORTANT NOTE:
We perform an initial quality control of all revised manuscripts before re-review. Your manuscript will FAIL this control and the handling will be delayed IN CASE the following APPLIES:

1) A data availability section providing access to data deposited in public databases is missing. If you have not deposited any data, please add a sentence to the data availability section that explains that.

2) Your manuscript contains statistics and error bars based on n=2. Please use scatter blots in these cases. No statistics should be calculated if n=2.

When submitting your revised manuscript, please carefully review the instructions that follow below. Failure to include requested items will delay the evaluation of your revision.*****

When submitting your revised manuscript, we will require:

1) a .docx formatted version of the manuscript text (including legends for main figures, EV figures and tables). Please make sure that the changes are highlighted to be clearly visible.

2) individual production quality figure files as .eps, .tif, .jpg (one file per figure).
Please download our Figure Preparation Guidelines (figure preparation pdf) from our Author Guidelines pages https://www.embopress.org/page/journal/14693178/authorguide for more info on how to prepare your figures.

3) a .docx formatted letter INCLUDING the reviewers' reports and your detailed point-by-point responses to their comments. As part of the EMBO Press transparent editorial process, the point-by-point response is part of the Review Process File (RPF), which will be published alongside your paper.

4) a complete author checklist, which you can download from our author guidelines (<https://www.embopress.org/page/journal/14693178/authorguide>). Please insert information in the checklist that is also reflected in the manuscript. The completed author checklist will also be part of the RPF.

5) Please note that all corresponding authors are required to supply an ORCID ID for their name upon submission of a revised manuscript (<https://orcid.org/>). Please find instructions on how to link your ORCID ID to your account in our manuscript tracking system in our Author guidelines (<https://www.embopress.org/page/journal/14693178/authorguide#authorshipguidelines>)

6) We replaced Supplementary Information with Expanded View (EV) Figures and Tables that are collapsible/expandable online. A maximum of 5 EV Figures can be typeset. EV Figures should be cited as 'Figure EV1, Figure EV2" etc... in the text and their respective legends should be included in the main text after the legends of regular figures.

- For the figures that you do NOT wish to display as Expanded View figures, they should be bundled together with their legends in a single PDF file called *Appendix*, which should start with a short Table of Content. Appendix figures should be referred to in the main text as: "Appendix Figure S1, Appendix Figure S2" etc. See detailed instructions regarding expanded view here: <https://www.embopress.org/page/journal/14693178/authorguide#expandedview>

- Additional Tables/Datasets should be labeled and referred to as Table EV1, Dataset EV1, etc. Legends have to be provided in a separate tab in case of .xls files. Alternatively, the legend can be supplied as a separate text file (README) and zipped together with the Table/Dataset file.

7) Please include a dedicated "Data Availability" section at the end of the Methods (suggested wording: "The [structural coordinates | microarray | mass spectrometry] data from this publication have been deposited to the [name of the database] database [URL] and assigned the identifier [accession | permalink | hashtag]."). Should this not apply, this should still be stated as "This study includes no data deposited in external repositories."

8) At EMBO Press we ask authors to provide source data for the main figures. Our source data coordinator will contact you to discuss which figure panels we would need source data for and will also provide you with helpful tips on how to upload and organize the files.

Additional information on source data and instruction on how to label the files are available <https://www.embopress.org/page/journal/14693178/authorguide#sourcedata>.

9) The journal requires a statement specifying whether or not authors have competing interests (defined as all potential or actual interests that could be perceived to influence the presentation or interpretation of an article). In case of competing interests, this must be specified in your disclosure statement. Further information: https://www.embopress.org/competing-interests

10) Figure legends and data quantification:
The following points must be specified in each figure legend:

- the name of the statistical test used to generate error bars and P values,
- the number (n) of independent experiments (please specify technical or biological replicates) underlying each data point,
- the nature of the bars and error bars (s.d., s.e.m.)

- If the data are obtained from n {less than or equal to} 5, show the individual data points in addition to the SD or SEM.
- If the data are obtained from n {less than or equal to} 2, use scatter blots showing the individual data points.

Discussion of statistical methodology can be reported in the materials and methods section, but figure legends should contain a basic description of n, P and the test applied.

See also the guidelines for figure legend preparation:
https://www.embopress.org/page/journal/14693178/authorguide#figureformat

- Please also include scale bars in all microscopy images and define their size in the legend, not the image.

11) Our journal encourages inclusion of *data citations in the reference list* to directly cite datasets that were re-used and obtained from public databases. Data citations in the article text are distinct from normal bibliographical citations and should directly link to the database records from which the data can be accessed. In the main text, data citations are formatted as follows: "Data ref: Smith et al, 2001" or "Data ref: NCBI Sequence Read Archive PRJNA342805, 2017". In the Reference list, data citations must be labeled with "[DATASET]". A data reference must provide the database name, accession number/identifiers and a resolvable link to the landing page from which the data can be accessed at the end of the reference. Further instructions are available at <https://www.embopress.org/page/journal/14693178/authorguide#referencesformat>.

12) All Materials and Methods need to be described in the main text using our 'Structured Methods' format. According to this format, the Methods section includes a Reagents and Tools Table (listing key reagents, experimental models, software and relevant equipment and including their sources and relevant identifiers) followed by a Methods and Protocols section describing the methods, ideally using a step-by-step protocol format. The aim is to facilitate adoption of the methodologies across labs. Please download and fill our Reagents and Tools Table template (.docx), which you can find in our author guidelines: https://www.embopress.org/page/journal/14693178/authorguide#structuredmethods.
When submitting your revised manuscript, please do not include the Reagents and Tools Table in the Methods section of the manuscript but upload it as a separate file choosing the file type "Reagent Table".
An example of a Method paper with Structured Methods can be found here:
https://www.embopress.org/doi/10.15252/msb.20178071.

13) As part of the EMBO publication's Transparent Editorial Process, EMBO Reports publishes online a Review Process File to accompany accepted manuscripts. This File will be published in conjunction with your paper and will include the referee reports,

your point-by-point response and all pertinent correspondence relating to the manuscript.

You are able to opt out of this by letting the editorial office know (emboreports@embo.org). If you do opt out, the Review Process File link will point to the following statement: "No Review Process File is available with this article, as the authors have chosen not to make the review process public in this case."

We would also welcome the submission of cover suggestions, or motifs to be used by our Graphics Illustrator in designing a cover.

I look forward to seeing a revised form of your manuscript when it is ready and please let me know if you have questions or comments regarding the revision.

Yours sincerely,

Martina Rembold, PhD
Senior Editor
EMBO reports

****

Referee #1:

In the manuscript titled "GEF14 acts as a specific activator of the plant osmotic signaling pathway by controlling ROP6 nanodomain formation," Gorgues et al. describe that ROP6 is part of the root response to osmotic stress. The authors also show the formation of ROP6 nanodomains at the membrane of epidermal cells, impacting the whole root response to osmotic stress. GEF14 is also found in nanodomains co-localizing with ROP6 during osmotic stress. GEF14 was previously proposed to be associated with ROP6, and the mutants show similar defects in ROS induction and whole root growth phenotypes during osmotic stress, while other GEF mutants do not. The paper reads well, is interesting, and is of high quality. However, the authors fail to convincingly show that GEF14 is indeed involved in the nanodomain recruitment for ROP6; most of the data suggest otherwise (experiments presented in figure 4 and 5). This is a major issue with the manuscript.
Major issues:

-Figure 1A is unclear to me as to which cells are shown; are they all epidermal cells? In the flagelin22 treatment for the rop6 mutants, it seems that the vasculature is the brightest, while in the control, just epidermal cells are shown. The same is true for Col-0 ABA treatment; it appears that the brightest fluorescent is coming from the vasculature, while in the mutant, it just comes from epidermal cells. Can the authors specify which cell types are being imaged? "Root cells" is a vague description. A similar observation for Figure 2, especially in 2C. The cells where ROS is activated are especially relevant if ROP6 and GEF14 are expressed mainly (or exclusively?) in epidermal cells (Figure 3). I realized this was published with similar pictures before, but the question stands and is a valid major concern. The authors must include BF images (or another contrasting stain) in the figure for further clarity.

-In line 172, the authors conclude:
"the level of ROS present in pROP6:cit-ROP6gCA expressing plants is higher than in wild type (9; Fig4 A). Interestingly, the level of ROS was comparable between pROP6:cit-ROP6gCA and gef14-2xpROP6:cit-ROP6gCA, and their fluorescent signal remained similar (Fig4 A, FigS4 A)."
However, the Col-0 pROP6:cit-ROP6g plants are not analyzed, so the authors cannot discard that the phenotypes are just linked to an overexpression of the protein in a background where the endogenous protein is also present. If the authors want to prove the statements in the cited text, they must show the ROS levels after osmotic stress in the gef14 2xpROP6:cit-ROP6g. The conclusion is valid if the ROS levels are not recovered to wild-type levels in those roots. This experimental result would also help make sense of the contradictory experiments presented in Figure 4 and strengthen the manuscript.
Furthermore, Figures 3A and B show that ROP6 is expressed in every epidermal cell, while GEF14 is expressed only in trichoblasts. The authors should analyze whether the ROP6 nanodomains are formed in response to osmotic stress in trichoblast and atrichoblasts, or only in trichoblasts where GEF14 is expressed. This result would also make the paper more convincing.
If the authors do not want to do extra experiments, I suggest modifying the conclusions to fit the data presented. The link between ROP6 nanodomain formation and osmotic stress is strong and would be an interesting and relevant paper to publish.

Referee #2:

In this study, the authors demonstrated that GEF14 acts as a specific activator of the plant osmotic signaling pathway by controlling ROP6 nanodomain formation. They found that ROP6 is not only required for osmotic and auxin responses, but is also involved in PAMPs and ABA signaling. In addition, the authors showed that ROP6 acts as a signaling hub, controlling different signaling pathways at the membrane, while GEF14 acts specifically in the osmotic signaling pathway. In addition, this study suggests that GEF14 can activate ROP6 by controlling the dynamics and clustering of ROP6, specifically in response to the osmotic signal. It is interesting and useful for understanding the role of Rho GTPases in response to environmental signals. I have a few suggestions that may improve their current work.

1. The authors' study showed that ROP6 is specifically involved in the osmotic response through GEF14. In the study, the authors looked at the response of ROP6 and GEF14 to osmosis by analyzing the level of ROS in roots only. Are there physiological phenotypes associated with rop6 and gef14 under osmotic stress? This is important to understand the role of ROP6 and GEF14 under osmotic stress.
2. Are there any defects in root hair length and density in rop6 and gef14?
3. Figure 1A, ROS levels in rop6-2 were significantly lower after ABA treatment than flg22 treatment, whereas the statistic in Figure 1B was that ROS levels were higher in rop6-2 after ABA treatment. Please verify that this is correct.
4. In Figure 2A, the wild-type ROS levels appear to be highest after osmotic treatment, but the statistics in Figure 2B show higher ROS levels in the complementation line. Please check the statistics for errors.
5. In Figure 5A, the cells shown in the GFP-ROP6 and gef14-2 x GFP-ROP6 materials do not look like the same type. Please check if the cell types are the same in both materials, otherwise, this comparison is meaningless.
6. The spelling of the mutant in the legend is not italicized, nor is the t before t-test. Please standardize the formatting.

Dear Editor,

Please find a revised version of our manuscript by Gorgues et al. We have now included a series of additional experiments in the manuscript. In particular, it now includes new data supporting the role of GEF14 in ROP6 clustering after osmotic stimulation. We also document the response of atrichoblastic cells where GEF14 is not strongly expressed. These new results demonstrate that both trichoblastic and atrichoblastic cells respond to osmotic stimulation in a GEF14-dependent manner. We hope these new experiments will fully address the questions raised by the reviewers

Sincerely

Alex, on behalf of the co-authors

Referee #1:

In the manuscript titled "GEF14 acts as a specific activator of the plant osmotic signaling pathway by controlling ROP6 nanodomain formation," Gorgues et al. describe that ROP6 is part of the root response to osmotic stress. The authors also show the formation of ROP6 nanodomains at the membrane of epidermal cells, impacting the whole root response to osmotic stress. GEF14 is also found in nanodomains co-localizing with ROP6 during osmotic stress. GEF14 was previously proposed to be associated with ROP6, and the mutants show similar defects in ROS induction and whole root growth phenotypes during osmotic stress, while other GEF mutants do not. The paper reads well, is interesting, and is of high quality.

However, the authors fail to convincingly show that GEF14 is indeed involved in the nanodomain recruitment for ROP6; most of the data suggest otherwise (experiments presented in figure 4 and 5). This is a major issue with the manuscript.

In previous study, our groups and others found that ROP6 formed nanodomains in the PM after cell stimulation (auxin and osmotic signal). To our knowledge, the most reliable experimental approach to quantify this effect is single molecule localization microscopy e.g. sptPALM on live root cells of Arabidopsis. Therefore, we used it to check the role of GEF14 on ROP6 nandomains formation in control condition, after osmotic and auxin stimuli. Our experiment show that the diffusion ROP6 molecules is reduced after an osmotic stimulation as previously documented (figure 5D, E) [1,2]. This effect is totally lost in *gef14-2* background (Figure 5 D, E). Such strong effect was previously shown in *feronia* and *pss1* mutant lines and was associated with the strong impairment in downstream signal processes [1,3]. Interestingly, the effect of the *gef14* mutant is specific to osmotic stimulation, since *gef14* has no impact on ROP6 dynamics in response to auxin (Figure 6D, E). This and other results presented in the manuscript bring us to propose that GEF14 is acting specifically in response to the osmotic signal.

To reinforce this idea, we also analyzed ROP6 clustering at the single molecule scale. These data showed that the local density of ROP6 molecules is lower in *gef14-2* background compare to control condition after an osmotic signal (Figure EV5 B, C). After a closer analysis of the data, it come from a smaller number of ROP6 molecules into ROP6 nanodomains in *gef14* mutant and is consistent with a model in which GEF14 is involved in the recruitment of ROP6 into nanodomains (Figure EV5 E). To confirm this result, we run a new set of experiments that is now included as supplemental figure EV5 G and H in the revise version of the manuscript. We quantified the GFP intensity within ROP6 nanodomains in the two following lines: GFP-ROP6 and *gef14-2*xGFP-ROP6. The TIRF data show that

pixel intensity in ROP6 nanodomains is smaller in *gef14-2* compare to Col0 after an osmotic stimulation (Figure EV5 G and H). We think these data are in lines with the results presented in the figure 5 and suggest that GEF14 is needed for the proper ROP6 nanodomains formation both in terms of diffusion and clustering.

Major issues:
-Figure 1A is unclear to me as to which cells are shown; are they all epidermal cells? In the flagelin22 treatment for the rop6 mutants, it seems that the vasculature is the brightest, while in the control, just epidermal cells are shown. The same is true for Col-0 ABA treatment; it appears that the brightest fluorescent is coming from the vasculature, while in the mutant, it just comes from epidermal cells. Can the authors specify which cell types are being imaged? "Root cells" is a vague description. A similar observation for Figure 2, especially in 2C. The cells where ROS is activated are especially relevant if ROP6 and GEF14 are expressed mainly (or exclusively?) in epidermal cells (Figure 3). I realized this was published with similar pictures before, but the question stands and is a valid major concern. The authors must include BF images (or another contrasting stain) in the figure for further clarity.

We totally agree with this comment and realized that our figures were not clearly representing the data in the graphs. Therefore, we prepared new images for the figure 1, 2 and EV1 where only the epidermal cells are shown. We also modified the text in the result and method sections to highlight that only epidermal cells were analyzed.

After running an initial test, we found that bright field images were not helping situating epidermal cells. Because DHE is staining the cell contour, we decided against including additional bright field images or contrasting stains, since individual cells can be readily detected in the DHE channel (see Figure 1A as an example). We believe the new version of the figures should now be much clearer, with regards to the cell analyzed.

-In line 172, the authors conclude:
"the level of ROS present in pROP6:cit-ROP6gCA expressing plants is higher than in wild type (9; Fig4 A). Interestingly, the level of ROS was comparable between pROP6:cit-ROP6gCA and gef14-2xpROP6:cit-ROP6gCA, and their fluorescent signal remained similar (Fig4 A, FigS4 A)."
However, the Col-0 pROP6:cit-ROP6g plants are not analyzed, so the authors cannot discard that the phenotypes are just linked to an overexpression of the protein in a background where the endogenous protein is also present. If the authors want to prove the statements in the cited text, they must show the ROS levels after osmotic stress in the gef14 2xpROP6:cit-ROP6g. The conclusion is valid if the ROS levels are not recovered to wild-type levels in those roots. This experimental result would also help make sense of the contradictory experiments presented in Figure 4 and strengthen the manuscript.

It was previously shown that the expression of ROP-CA (G15V) point mutant in root and petals lead to high ROS accumulation [4–6]. We later confirmed this observation and showed this was not due to over accumulation of the protein in epidermal cells ([1] see figS3 E and F). To further verify this observation, we run a new series of experiments (Figure1 of the response to reviewer comments). In this experiment, the citrine fluorescent signal between pROP6:cit-ROP6g and pROP6:cit-ROP6gCA is similar (Figure1 A of the response to reviewer comments). Then, we quantified the DHE signal in Col-0, pROP6:cit-ROP6g and pROP6:cit-ROP6gCA and found that pROP6:cit-ROP6gCA has a higher DHE

signal (Figure1 B of the response to reviewer comments). This result suggests that the high ROS accumulation comes from the expression of ROP6 in its lock version (ROP6CA) rather than an effect on protein accumulation.

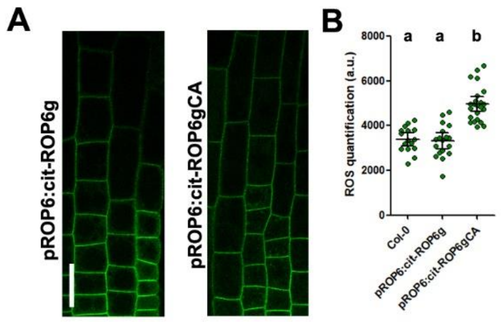

Figure 1: (A) Confocal image of pROP6:Cit-ROP6g and pROP6:Cit-ROP6gCA. (B) Quantification of DHE staining of Col-0, pROP6:Cit-ROP6g and pROP6:Cit-ROP6gCA epidermal cells. Scale bar 10 µm, n>16 from two independent biological replicas. ANOVA followed by a post hoc Tukey test. Letters represent differences with a p-value bellow 0.05.

According to this result, we believed that comparing pROP6:cit-ROP6gCA to *gef14-2*xpROP6:cit-ROP6gCA is enough to conclude that GEF14 is not needed for ROS induce by ROP6-CA. The text in the result section was modified to explain in clearer terms our conclusion.

Furthermore, Figures 3A and B show that ROP6 is expressed in every epidermal cell, while GEF14 is expressed only in trichoblasts. The authors should analyze whether the ROP6 nanodomains are formed in response to osmotic stress in trichoblast and atrichoblasts, or only in trichoblasts where GEF14 is expressed. This result would also make the paper more convincing.
If the authors do not want to do extra experiments, I suggest modifying the conclusions to fit the data presented. The link between ROP6 nanodomain formation and osmotic stress is strong and would be an interesting and relevant paper to publish.

This is an interesting remark. We reran the experiments to directly analyze the ROP6 localization in atrichoblast cells. Interestingly, we found that atrichoblast cells form ROP6 nanodomain in a GEF14-dependent manner (Figure 5G, H). This result suggests that atrichoblasts can react to osmotic stimulation in a non-cell autonomous way. Alternatively, we cannot exclude that GEF14 is present at a low level in atrichoblasts, below our detection sensitivity, but sufficiently to trigger ROP6 activation. We have now included this data and conclusion in the revised manuscript.

Referee #2:

In this study, the authors demonstrated that GEF14 acts as a specific activator of the plant osmotic signaling pathway by controlling ROP6 nanodomain formation. They found that ROP6 is not only required for osmotic and auxin responses, but is also involved in PAMPs and ABA signaling. In addition, the authors showed that ROP6 acts as a signaling hub, controlling different signaling pathways at the membrane, while GEF14 acts specifically in the osmotic signaling pathway. In addition, this study suggests that GEF14 can activate ROP6 by controlling the dynamics and clustering of ROP6, specifically in response to the osmotic signal. It is interesting and useful for understanding the role of Rho GTPases in response to environmental signals. I have a few suggestions that may improve their current work.

1. The authors' study showed that ROP6 is specifically involved in the osmotic response through GEF14. In the study, the authors looked at the response of ROP6 and GEF14 to osmosis by analyzing the level of ROS in roots only. Are there physiological phenotypes associated with rop6 and gef14

under osmotic stress? This is important to understand the role of ROP6 and GEF14 under osmotic stress.

We thank the reviewer 2 for this comment. Analysis in our root phenotypical platform show that the overall root architecture is not revealing any striking differences between genotype or treatment. We only identify a slight and not significant primary root growth effect (Figure EV5 I, J, K). In a recent report, it was shown that lateral root initiation is repress by strong osmotic challenges [7]. Therefore, we quantify the density of primordia in the root exposed to osmotic stress. First, we found that *rbohD/F*, that accumulate less osmotically-induced ROS [8], has more primordia compare to Col-0 plant under stress condition (Figure EV5 L). This result suggests that ROS is a negative regulator of primordia initiation. Interestingly, a very similar observation is reported in a preprint. In this article, ROS mediated oligomerization of IAA3/SHY2 is required for efficient ARF mediated target gene repression during inhibition of lateral root by the osmotic signal [9]. Interestingly, our experiment also shows that *rop6-2* and *gef14-2* present a similar phenotype than *rbohD/F* under stress condition (Figure EV5 L). Therefore, we speculate that activation of the GEF14/ROP6/RBOHD/F module by osmotic signal, lead to an inhibition of primordia initiation. Even if it is still speculative at this stage, we decided to discuss this model in the discussion.

2. Are there any defects in root hair length and density in rop6 and gef14?

We thank the reviewer for this comment. Indeed, several ROPs and GEFs are known to regulate root hair development [10–12]. We therefore conducted experiments to test the role of ROP6 and GEF14 in this process. Whereas, *rop6-2* show a mild default in root hair density, distance to the first bulge and length, the two GEF14 mutant lines does not. This result suggests that ROP6 effect on root hair is not mediated by GEF14. The data are now included in the Figure EV6 C, D, E, F. The results and the methods section was modify accordingly.

3. Figure 1A, ROS levels in rop6-2 were significantly lower after ABA treatment than flg22 treatment, whereas the statistic in Figure 1B was that ROS levels were higher in rop6-2 after ABA treatment. Please verify that this is correct.

We apologized for this and similar observation was raised by the reviewer1. Therefore, we now remade the figure 1, 2 and Figure EV1 to avoid any confusion. Indeed, data quantification was made only in root epidermal cells. We hope that the new figures will clarify this point.

4. In Figure 2A, the wild-type ROS levels appear to be highest after osmotic treatment, but the statistics in Figure 2B show higher ROS levels in the complementation line. Please check the statistics for errors.

Thank you for this remark. See our response in the point 3.

5. In Figure 5A, the cells shown in the GFP-ROP6 and gef14-2 x GFP-ROP6 materials do not look like the same type. Please check if the cell types are the same in both materials, otherwise, this comparison is meaningless.

We totally agree, the test was made on comparable cells. We change the picture to illustrate the graph (Figure 5 A).

6. The spelling of the mutant in the legend is not italicized, nor is the t before t-test. Please standardize the formatting.

Thank you for the remarks. It is now corrected

References

1. Smokvarska, M., Francis, C., Platre, M.P., Fiche, J.-B., Alcon, C., Dumont, X., Nacry, P., Bayle, V., Nollmann, M., Maurel, C., et al. (2020). A Plasma Membrane Nanodomain Ensures Signal Specificity during Osmotic Signaling in Plants. Curr Biol *30*, 4654-4664.e4. https://doi.org/10.1016/j.cub.2020.09.013.

2. Smokvarska, M., Bayle, V., Maneta-Peyret, L., Fouillen, L., Poitout, A., Dongois, A., Fiche, J.-B., Gronnier, J., Garcia, J., Höfte, H., et al. (2023). The receptor kinase FERONIA regulates phosphatidylserine localization at the cell surface to modulate ROP signaling. Sci Adv *9*, eadd4791. https://doi.org/10.1126/sciadv.add4791.

3. Platre, M.P., Bayle, V., Armengot, L., Bareille, J., Marquès-Bueno, M.D.M., Creff, A., Maneta-Peyret, L., Fiche, J.-B., Nollmann, M., Miège, C., et al. (2019). Developmental control of plant Rho GTPase nano-organization by the lipid phosphatidylserine. Science *364*, 57–62. https://doi.org/10.1126/science.aav9959.

4. Duan, Q., Kita, D., Li, C., Cheung, A.Y., and Wu, H.-M. (2010). FERONIA receptor-like kinase regulates RHO GTPase signaling of root hair development. Proc. Natl. Acad. Sci. U.S.A. *107*, 17821–17826. https://doi.org/10.1073/pnas.1005366107.

5. Sorek, N., Segev, O., Gutman, O., Bar, E., Richter, S., Poraty, L., Hirsch, J.A., Henis, Y.I., Lewinsohn, E., Jürgens, G., et al. (2010). An S-acylation switch of conserved G domain cysteines is required for polarity signaling by ROP GTPases. Curr. Biol. *20*, 914–920. https://doi.org/10.1016/j.cub.2010.03.057.

6. Dang, X., Yu, P., Li, Y., Yang, Y., Zhang, Y., Ren, H., Chen, B., and Lin, D. (2018). Reactive oxygen species mediate conical cell shaping in Arabidopsis thaliana petals. PLoS Genet. *14*, e1007705. https://doi.org/10.1371/journal.pgen.1007705.

7. Mehra, P., Pandey, B.K., Melebari, D., Banda, J., Leftley, N., Couvreur, V., Rowe, J., Anfang, M., De Gernier, H., Morris, E., et al. (2022). Hydraulic flux-responsive hormone redistribution determines root branching. Science *378*, 762–768. https://doi.org/10.1126/science.add3771.

8. Martinière, A., Fiche, J.B., Smokvarska, M., Mari, S., Alcon, C., Dumont, X., Hematy, K., Jaillais, Y., Nollmann, M., and Maurel, C. (2019). Osmotic Stress Activates Two Reactive Oxygen Species Pathways with Distinct Effects on Protein Nanodomains and Diffusion. Plant Physiol. *179*, 1581–1593. https://doi.org/10.1104/pp.18.01065.

9. Roy, D., Mehra, P., Mukkawar, V., Clark, L., Bellande, K., Vermeer, J.E., Arevallilo, R.M., Vernoux, T., Ob, K.S., Jones, A., et al. (2024). ROS responsive Aux/IAA multimerization modulates auxin responses. Preprint at bioRxiv, https://doi.org/10.1101/2024.02.12.579961 https://doi.org/10.1101/2024.02.12.579961.

10. Denninger, P., Reichelt, A., Schmidt, V.A.F., Mehlhorn, D.G., Asseck, L.Y., Stanley, C.E., Keinath, N.F., Evers, J.-F., Grefen, C., and Grossmann, G. (2019). Distinct RopGEFs Successively Drive

Polarization and Outgrowth of Root Hairs. Curr. Biol. *29*, 1854-1865.e5. https://doi.org/10.1016/j.cub.2019.04.059.

11. Gendre, D., Baral, A., Dang, X., Esnay, N., Boutté, Y., Stanislas, T., Vain, T., Claverol, S., Gustavsson, A., Lin, D., et al. (2019). Rho-of-plant activated root hair formation requires Arabidopsis YIP4a/b gene function. Development *146*. https://doi.org/10.1242/dev.168559.

12. Hirano, T., Konno, H., Takeda, S., Dolan, L., Kato, M., Aoyama, T., Higaki, T., Takigawa-Imamura, H., and Sato, M.H. (2018). PtdIns(3,5)P 2 mediates root hair shank hardening in Arabidopsis. Nature Plants *4*, 888–897. https://doi.org/10.1038/s41477-018-0277-8.

1st Revision - Editorial Decision **29th Jan 2025**

Dear Dr. Martinière

Thank you for the submission of your revised manuscript to EMBO reports. We have now received the full set of referee reports that is copied below.

As you will see, both referees are very positive about the study and recommend publication. Before I can accept the manuscript, I need you to address some minor points below:

- Please label the corresponding author on the manuscript title page and provide the e-mail address.

- Please provide up to 5 keywords.

- Please reformat the references. They should be alphabetical, not numerical. The DOI is only used for preprint citations or references to datasets (see also the next point).

- Please use our format for citing preprint articles. The in-text citation should be (preprint: name et al, year). In the reference list please add [PREPRINT] to the reference, like this: Author Name1, Author Name2 etc (year) article title. biorXiv doi [PREPRINT].

- Panel (F) is skipped in Figure 5.

- Author Checklist: please complete the top left general info table and the section "Experimental animals". You can choose Not Applicable here.

- You indicate in the Author Checklist that you included a statement on blinding, but I could not find it. Please check.

- Please upload the figures as individual high-resolution files with the figure legends removed. These should only be present in the manuscript file.

- Please change the nomenclature for EV figures to Figure EVx in the EV figure legends.

- Please provide a Reagents and Tools Table, listing key reagents, experimental models, software and relevant equipment and including their sources and relevant identifiers. You can download the template (.docx), from our author guidelines: https://www.embopress.org/page/journal/14693178/authorguide#structuredmethods.
When submitting your revised manuscript, please do not include the Reagents and Tools Table in the Methods section of the manuscript but upload it as a separate file choosing the file type "Reagent Table".
An example of a Method paper with Structured Methods can be found here: https://www.embopress.org/doi/10.15252/msb.20178071.

- The section order should be corrected: Title page - Abstract & Keywords - Introduction - Results - Discussion - Methods - Data Availability - Acknowledgements - Disclosure and Competing Interests Statement - References - Figure Legends - Table(s) - Expanded View Figure Legends.

- Our production/data editors have asked you to clarify several points in the figure legends (see below). Please incorporate these changes in the manuscript and return the revised file with tracked changes with your final manuscript submission.

A) Statistical test information. Only p-values that are actually shown in the figure panel(s) should (and must) be defined in the legends, all others should be removed from (or added to) the legend. Moreover, we ask for the specification of exact p-values:
- Please define the annotated p values ****/***/**/* as well as provide the exact p-values for the same in the legend of figure EV3 D, EV5 H as appropriate.
- Please note that the exact p values are not provided in the legends of figures 1C, D; 2B, D; 4A, D, E; 5D, E, H; 6B, E, G; EV1 B, EV2 A, B; EV5 C, D, E, L; EV6 B, D, E, F.

B) Replicates and error bars:
- Please note that the error bars are not defined in the legend of figure EV3 D.
- Please note that the measure of center for the error bars needs to be defined in the legends of figures 1C, D; 2B, D; 3E, F; 4A, D, E, G; 5A, D, E, G, H; 6B, C, D, E, G; EV1 B, C; EV2 A, B; EV3 D, EV5 B, C, D, E, F, H, L.

C) Data presentation:
- Please note that the scale bar needs to be defined for figure 3G
- Please note that the white arrows are not defined in the legend of figure EV3 A. This needs to be rectified.

- When you determine statistical significance based on the mean for n cells measured in N independent experiments (pooled),

would that not represent sort of a pseudoreplication as opposed to calculating statistical significance based on the mean of the means of each condition? Or am I mistaken?

- As a standard procedure, we edit the title and abstract of manuscripts to make them more accessible to a general readership. Please find the edited draft below my signature.

- Finally, EMBO Reports papers are accompanied online by
A) a short (1-2 sentences) summary of the findings and their significance,
B) 2-3 bullet points highlighting key results and
C) a schematic summary figure that provides a sketch of the major findings (not a data image).
Please provide the summary figure as a separate file in PNG or JPG format at a size of 550x300-600 pixels (width x height). Please note that the size is rather small and that text needs to be readable at the final size. Please send us this information along with the revised manuscript.

- On a different note, I would like to alert you that EMBO Press offers a new format for a video-synopsis of work published with us, which essentially is a short, author-generated film explaining the core findings in hand drawings, and, as we believe, can be very useful to increase visibility of the work. This has proven to offer a nice opportunity for exposure i.p. for the first author(s) of the study. Please see the following link for representative examples and their integration into the article web page:
https://www.embopress.org/video_synopses
https://www.embopress.org/doi/full/10.15252/embj.2019103932

Please let me know, should you be interested to engage in commissioning a similar video synopsis for your work. According operation instructions are available and intuitive.

We look forward to seeing a final version of your manuscript as soon as possible.

With kind regards,

Martina Rembold, PhD
Senior Editor
EMBO reports

=================

Referee #1:

The authors have addressed all my comments.

Referee #2:

The authors have answered all the questions raised by the reviewer very well, and I believe the manuscript can now be published.

==================

Suggestions for the title (max. 100 characters incl. spaces):

GEF14 activates the plant osmotic signaling pathway and promotes ROP6 nanodomain formation
GEF14 is an osmotic stress-specific activator of ROP6
GEF14 promotes ROP6 nanodomain formation in response to osmotic stress.

Abstract

During their growth, plants encounter and respond to a variety of environmental signals. However, the mechanisms underlying the integration and specificity of signals remain poorly understood. Rho of Plant (ROP) signaling plays a central role in various processes, including polar cell growth and responses to different stimuli, and relies on stimuli-dependent membrane nanodomains. The effector composition of ROP6 nanodomains varies depending on the signal and may be involved in downstream signal specificity. In this study, we explore how ROP6 signaling is regulated by Guanine nucleotide Exchange Factor (GEF) during osmotic stress. We find that GEF14 is required for osmotically induced ROS accumulation. This isoform

acts specifically in response to osmotic stimulation, since it is dispensable for other stimuli. We demonstrate that GEF14 activates ROP6 and controls its clustering in a signal-specific manner. Furthermore, we find that GEF14 relocates from the cytoplasm to clusters at the plasma membrane after osmotic stimulation. Together, our results suggest that a single GEF isoform can encode for signal specificity controlling ROP6 activation, clustering and downstream cellular responses.

Dear Martina Rembold,

Thank you for this great news. We are very happy to have our work published in EMBO reports. We also thank you for the details reading of the work and the different point raised. Please find below our responses.

Please do not hesitate to contact me if you need anything more.

Sincerely,

Alex

Dear Dr. Martinière

Thank you for the submission of your revised manuscript to EMBO reports. We have now received the full set of referee reports that is copied below.

As you will see, both referees are very positive about the study and recommend publication. Before I can accept the manuscript, I need you to address some minor points below:

- Please label the corresponding author on the manuscript title page and provide the e-mail address.

>OK

- Please provide up to 5 keywords.

>OK

- Please reformat the references. They should be alphabetical, not numerical. The DOI is only used for preprint citations or references to datasets (see also the next point).

>OK

- Please use our format for citing preprint articles. The in-text citation should be (preprint: name et al, year). In the reference list please add [PREPRINT] to the reference, like this: Author Name1, Author Name2 etc (year) article title. biorXiv doi [PREPRINT].

>OK

- Panel (F) is skipped in Figure 5.

>OK

- Author Checklist: please complete the top left general info table and the section "Experimental animals". You can choose Not Applicable here.
>OK

- You indicate in the Author Checklist that you included a statement on blinding, but I could not find it. Please check.

> It is in the section on statistics

- Please upload the figures as individual high-resolution files with the figure legends removed. These should only be present in the manuscript file.

>OK

- Please change the nomenclature for EV figures to Figure EVx in the EV figure legends.

>OK

- Please provide a Reagents and Tools Table, listing key reagents, experimental models, software and relevant equipment and including their sources and relevant identifiers. You can download the template (.docx), from our author guidelines:
https://www.embopress.org/page/journal/14693178/authorguide#structuredmethods.
When submitting your revised manuscript, please do not include the Reagents and Tools Table in the Methods section of the manuscript but upload it as a separate file choosing the file type "Reagent Table".
An example of a Method paper with Structured Methods can be found here:
https://www.embopress.org/doi/10.15252/msb.20178071.

>OK

- The section order should be corrected: Title page - Abstract & Keywords - Introduction - Results - Discussion - Methods - Data Availability - Acknowledgements - Disclosure and Competing Interests Statement - References - Figure Legends - Table(s) - Expanded View Figure Legends.

>OK

- Our production/data editors have asked you to clarify several points in the figure legends (see below). Please incorporate these changes in the manuscript and return the revised file with tracked changes with your final manuscript submission.

A) Statistical test information. Only p-values that are actually shown in the figure panel(s) should (and must) be defined in the legends, all others should be removed from (or added to) the legend. Moreover, we ask for the specification of exact p-values:
- Please define the annotated p values ****/***/**/* as well as provide the exact p-values for the same in the legend of figure EV3 D, EV5 H as appropriate.

>OK this is now in the legends

- Please note that the exact p values are not provided in the legends of figures 1C, D; 2B, D; 4A, D, E; 5D, E, H; 6B, E, G; EV1 B, EV2 A, B; EV5 C, D, E, L; EV6 B, D, E, F.

>The multiple comparison of means was performed using the Tukey test. The letters indicate significant differences with a threshold of p-value < 0.05. Adding the exact p-value for each comparison would be too extensive and likely not helpful to readers. For the t-test, we provide the exact p-value.

B) Replicates and error bars:
- Please note that the error bars are not defined in the legend of figure EV3 D.
- Please note that the measure of center for the error bars needs to be defined in the legends of figures 1C, D; 2B, D; 3E, F; 4A, D, E, G; 5A, D, E, G, H; 6B, C, D, E, G; EV1 B, C; EV2 A, B; EV3 D, EV5 B, C, D, E, F, H, L.
>OK

C) Data presentation:
- Please note that the scale bar needs to be defined for figure 3G

>OK

- Please note that the white arrows are not defined in the legend of figure EV3 A. This needs to be rectified.
>OK

- When you determine statistical significance based on the mean for n cells measured in N independent experiments (pooled), would that not represent sort of a pseudoreplication as opposed to calculating statistical significance based on the mean of the means of each condition? Or am I mistaken?

> In a typical experiment, we measure 9 to 12 cells. This measurement is repeated in different plants, generally between 5 and 7. The whole procedure is repeated two to three times, and we carry out statistical tests to verify the observed effects. When the observations are consistent and because we have observed that the variability between cells of the same plant is often greater than the variability between different plants, all the data is pooled. Thus, I do not think we are doing repetitive measurements, e.g., pseudoreplicates.

- As a standard procedure, we edit the title and abstract of manuscripts to make them more accessible to a general readership. Please find the edited draft below my signature.

>The revised abstract is perfect. I have pasted it in the manuscript.

- Finally, EMBO Reports papers are accompanied online by
A) a short (1-2 sentences) summary of the findings and their significance,

>I have add it to the manuscript after the keywords

B) 2-3 bullet points highlighting key results and

>Rho Of Plant 6 (ROP6) is a hub for membrane signaling

>Guanine nucleotide Exange Factor 14 (GEF14) confer osmotic signaling specificity

>GEF14 regulates ROP6 nanodomains formation during osmotic signaling

C) a schematic summary figure that provides a sketch of the major findings (not a data image).
Please provide the summary figure as a separate file in PNG or JPG format at a size of 550x300-600

pixels (width x height). Please note that the size is rather small and that text needs to be readable at the final size. Please send us this information along with the revised manuscript.

>Uploaded on the EMBO webpage

- On a different note, I would like to alert you that EMBO Press offers a new format for a video-synopsis of work published with us, which essentially is a short, author-generated film explaining the core findings in hand drawings, and, as we believe, can be very useful to increase visibility of the work. This has proven to offer a nice opportunity for exposure i.p. for the first author(s) of the study. Please see the following link for representative examples and their integration into the article web page:
https://www.embopress.org/video_synopses
https://www.embopress.org/doi/full/10.15252/embj.2019103932

Please let me know, should you be interested to engage in commissioning a similar video synopsis for your work. According operation instructions are available and intuitive.

We look forward to seeing a final version of your manuscript as soon as possible.

With kind regards,

Martina Rembold, PhD
Senior Editor
EMBO reports

=================

Referee #1:

The authors have addressed all my comments.

Referee #2:

The authors have answered all the questions raised by the reviewer very well, and I believe the manuscript can now be published.

==================

Suggestions for the title (max. 100 characters incl. spaces):

GEF14 activates the plant osmotic signaling pathway by promoting the formation of ROP6 nanodomain

Abstract

During their growth, plants encounter and respond to a variety of environmental signals. However, the mechanisms underlying the integration and specificity of signals remain poorly understood. Rho of Plant (ROP) signaling plays a central role in various processes, including polar cell growth and responses to different stimuli, and relies on stimuli-dependent membrane nanodomains. The effector composition of ROP6 nanodomains varies depending on the signal and may be involved in downstream signal specificity. In this study, we explore how ROP6 signaling is regulated by Guanine nucleotide Exchange Factor (GEF) during osmotic stress. We find that GEF14 is required for osmotically induced ROS accumulation. This isoform acts specifically in response to osmotic stimulation, since it is dispensable for other stimuli. We demonstrate that GEF14 activates ROP6 and controls its clustering in a signal-specific manner. Furthermore, we find that GEF14 relocates from the cytoplasm to clusters at the plasma membrane after osmotic stimulation. Together, our results suggest that a single GEF isoform can encode for signal specificity controlling ROP6 activation, clustering and downstream cellular responses.

Alexandre Martinière
CNRS/INRAE
Institute for Plant Sciences of Montpellier (IPSiM)
Campus INRAE/Institut Agro
2 place Viala
Montpellier Cedex 1 F-34060
France

Dear Dr. Martinière,

I am very pleased to accept your manuscript for publication in the next available issue of EMBO reports. Thank you for your contribution to our journal.

Your manuscript will be processed for publication by EMBO Press. It will be copy edited and you will receive page proofs prior to publication. Please note that you will be contacted by Springer Nature Author Services to complete licensing and payment information.

You may qualify for financial assistance for your publication charges - either via a Springer Nature fully open access agreement or an EMBO initiative. Check your eligibility: https://www.embopress.org/page/journal/14693178/authorguide#chargesguide

Should you be planning a Press Release on your article, please get in contact with embo_production@springernature.com as early as possible in order to coordinate publication and release dates.

If you have any questions, please do not hesitate to contact the Editorial Office. Thank you for your contribution to EMBO Reports.

Kind regards,

Martina Rembold, PhD
Senior Editor
EMBO reports

>>> Please note that it is EMBO Reports policy for the transcript of the editorial process (containing referee reports and your response letter) to be published as an online supplement to each paper. If you do NOT want this, you will need to inform the Editorial Office via email immediately. More information is available here: https://www.embopress.org/transparent-process#Review_Process

EMBO Press Author Checklist

Corresponding Author Name: Martiniere Alexandre
Journal Submitted to: EMBO report
Manuscript Number: EMBOR-2024-60147V2

USEFUL LINKS FOR COMPLETING THIS FORM
The EMBO Journal - Author Guidelines
EMBO Reports - Author Guidelines
Molecular Systems Biology - Author Guidelines
EMBO Molecular Medicine - Author Guidelines

Reporting Checklist for Life Science Articles (updated January
This checklist is adapted from Materials Design Analysis Reporting (MDAR) Checklist for Authors. MDAR establishes a minimum set of requirements in transparent reporting in the life sciences (see Statement of Task: 10.31222/osf.io/9sm4x). Please follow the journal's guidelines in preparing your
Please note that a copy of this checklist will be published alongside your article.

Abridged guidelines for figures
1. Data
The data shown in figures should satisfy the following conditions:
- the data were obtained and processed according to the field's best practice and are presented to reflect the results of the experiments in an accurate and unbiased manner.
- ideally, figure panels should include only measurements that are directly comparable to each other and obtained with the same assay.
- plots include clearly labeled error bars for independent experiments and sample sizes. Unless justified, error bars should not be shown for technical
- if n<5, the individual data points from each experiment should be plotted. Any statistical test employed should be justified.
- Source Data should be included to report the data underlying figures according to the guidelines set out in the authorship guidelines on Data

2. Captions
Each figure caption should contain the following information, for each panel where they are relevant:
- a specification of the experimental system investigated (eg cell line, species name).
- the assay(s) and method(s) used to carry out the reported observations and measurements.
- an explicit mention of the biological and chemical entity(ies) that are being measured.
- an explicit mention of the biological and chemical entity(ies) that are altered/varied/perturbed in a controlled manner.
- the exact sample size (n) for each experimental group/condition, given as a number, not a range;
- a description of the sample collection allowing the reader to understand whether the samples represent technical or biological replicates (including how many animals, litters, cultures, etc.).
- a statement of how many times the experiment shown was independently replicated in the laboratory.
- definitions of statistical methods and measures:
  - common tests, such as t-test (please specify whether paired vs. unpaired), simple χ2 tests, Wilcoxon and Mann-Whitney tests, can be unambiguously identified by name only, but more complex techniques should be described in the methods section;
  - are tests one-sided or two-sided?
  - are there adjustments for multiple comparisons?
  - exact statistical test results, e.g., P values = x but not P values < x;
  - definition of 'center values' as median or average;
  - definition of error bars as s.d. or s.e.m.

Please complete ALL of the questions below.
Select "Not Applicable" only when the requested information is not relevant for your study.

Materials

Newly Created Materials	Information included in the manuscript?	In which section is the information available? (Reagents and Tools Table, Materials and Methods, Figures, Data Availability Section)
New materials and reagents need to be available; do any restrictions apply?	Yes	Materials and Methods

Antibodies	Information included in the manuscript?	In which section is the information available? (Reagents and Tools Table, Materials and Methods, Figures, Data Availability Section)
For **antibodies** provide the following information:<br>- Commercial antibodies: RRID (if possible) or supplier name, catalogue number and or/clone number<br>- Non-commercial: RRID or citation	Yes	Materials and Methods

DNA and RNA sequences	Information included in the manuscript?	In which section is the information available? (Reagents and Tools Table, Materials and Methods, Figures, Data Availability Section)
**Short novel DNA or RNA including primers, probes**: provide the sequences.	Not Applicable	

Cell materials	Information included in the manuscript?	In which section is the information available? (Reagents and Tools Table, Materials and Methods, Figures, Data Availability Section)
**Cell lines:** Provide species information, strain. Provide accession number in repository **OR** supplier name, catalog number, clone number, and/**OR** RRID.	Not Applicable	
**Primary cultures:** Provide species, strain, sex of origin, genetic modification status.	Not Applicable	
Report if the cell lines were recently **authenticated** (e.g., by STR profiling) and tested for mycoplasma contamination.	Not Applicable	

Experimental animals	Information included in the manuscript?	In which section is the information available? (Reagents and Tools Table, Materials and Methods, Figures, Data Availability Section)
**Laboratory animals or Model organisms:** Provide species, strain, sex, age, genetic modification status. Provide accession number in repository **OR** supplier name, catalog number, clone number, **OR** RRID.	Not Applicable	
**Animal observed in or captured from the field:** Provide species, sex, and age where possible.	Not Applicable	
Please detail **housing and husbandry conditions**.	Not Applicable	

Plants and microbes	Information included in the manuscript?	In which section is the information available? (Reagents and Tools Table, Materials and Methods, Figures, Data Availability Section)
**Plants:** provide species and strain, ecotype and cultivar where relevant, unique accession number if available, and source (including location for collected wild specimens).	Yes	Materials and Methods
**Microbes:** provide species and strain, unique accession number if available, and source.	Not Applicable	

Human research participants	Information included in the manuscript?	In which section is the information available? (Reagents and Tools Table, Materials and Methods, Figures, Data Availability Section)
If collected and within the bounds of privacy constraints report on age, sex and gender or ethnicity for all study participants.	Not Applicable	

Core facilities	Information included in the manuscript?	In which section is the information available? (Reagents and Tools Table, Materials and Methods, Figures, Data Availability Section)
If your work benefited from core facilities, was their service mentioned in the acknowledgments section?	Not Applicable	

Design

Study protocol	Information included in the manuscript?	In which section is the information available? (Reagents and Tools Table, Materials and Methods, Figures, Data Availability Section)
If study protocol has been **pre-registered, provide DOI in the manuscript**. For clinical trials, provide the trial registration number **OR** cite DOI.	Not Applicable	
Report the **clinical trial registration number** (at ClinicalTrials.gov or equivalent), where applicable.	Not Applicable	

Laboratory protocol	Information included in the manuscript?	In which section is the information available? (Reagents and Tools Table, Materials and Methods, Figures, Data Availability Section)
Provide DOI OR other citation details if **external detailed step-by-step protocols** are available.	Yes	Materials and Methods

Experimental study design and statistics	Information included in the manuscript?	In which section is the information available? (Reagents and Tools Table, Materials and Methods, Figures, Data Availability Section)
Include a statement about **sample size** estimate even if no statistical methods were used.	Yes	Materials and Methods, Figures
Were any steps taken to minimize the effects of subjective bias when allocating animals/samples to treatment (e.g. **randomization procedure**)? If yes, have they been described?	Not Applicable	
Include a statement about **blinding** even if no blinding was done.	Yes	Materials and Methods
Describe **inclusion/exclusion criteria** if samples or animals were excluded from the analysis. Were the criteria pre-established?<br><br>If sample or data points were omitted from analysis, report if this was due to attrition or intentional exclusion and provide justification.	Not Applicable	
For every figure, are **statistical tests** justified as appropriate? Do the data meet the assumptions of the tests (e.g., normal distribution)? Describe any methods used to assess it. Is there an estimate of variation within each group of data? Is the variance similar between the groups that are being statistically compared?	Yes	Materials and Methods

Sample definition and in-laboratory replication	Information included in the manuscript?	In which section is the information available? (Reagents and Tools Table, Materials and Methods, Figures, Data Availability Section)
In the figure legends: state number of times the experiment was **replicated** in laboratory.	Yes	Materials and Methods, Figures
In the figure legends: define whether data describe **technical or biological replicates**.	Yes	Materials and Methods, Figures

Ethics

Ethics	Information included in the manuscript?	In which section is the information available? (Reagents and Tools Table, Materials and Methods, Figures, Data Availability Section)
Studies involving **human participants**: State details of **authority granting ethics approval** (IRB or equivalent committee(s), provide reference number for approval.	Not Applicable	
Studies involving **human participants**: Include a statement confirming that **informed consent** was obtained from all subjects and that the experiments conformed to the principles set out in the WMA Declaration of Helsinki and the Department of Health and Human Services Belmont Report.	Not Applicable	
Studies involving **human participants:** For publication of **patient photos**, include a statement confirming that consent to publish was obtained.	Not Applicable	
Studies involving experimental **animals**: State details of **authority granting ethics approval** (IRB or equivalent committee(s), provide reference number for approval. Include a statement of compliance with ethical regulations.	Not Applicable	
Studies involving **specimen and field samples:** State if relevant **permits** obtained, provide details of authority approving study; if none were required, explain why.	Not Applicable	

Dual Use Research of Concern (DURC)	Information included in the manuscript?	In which section is the information available? (Reagents and Tools Table, Materials and Methods, Figures, Data Availability Section)
Could your study fall under dual use research restrictions? Please check biosecurity documents and list of **select agents and toxins** (CDC): https://www.selectagents.gov/sat/list.htm	Not Applicable	
If you used a select agent, is the security level of the lab appropriate and reported in the manuscript?	Not Applicable	
If a study is subject to dual use research of concern regulations, is the name of the **authority granting approval and reference number** for the regulatory approval provided in the manuscript?	Not Applicable	

Reporting

The MDAR framework recommends adoption of discipline-specific guidelines, established and endorsed through community initiatives. Journals have their own policy about requiring specific guidelines and recommendations to complement MDAR.

Adherence to community standards	Information included in the manuscript?	In which section is the information available? (Reagents and Tools Table, Materials and Methods, Figures, Data Availability Section)
State if relevant guidelines or checklists (e.g., **ICMJE, MIBBI, ARRIVE, PRISMA**) have been followed or provided.	Not Applicable	
For **tumor marker prognostic studies,** we recommend that you follow the **REMARK** reporting guidelines (see link list at top right). See author guidelines, under 'Reporting Guidelines'. Please confirm you have followed these guidelines.	Not Applicable	
For **phase II and III randomized controlled trials**, please refer to the **CONSORT** flow diagram (see link list at top right) and submit the CONSORT checklist (see link list at top right) with your submission. See author guidelines, under 'Reporting Guidelines'. Please confirm you have submitted this list.	Not Applicable	

Data Availability

Data availability	Information included in the manuscript?	In which section is the information available? (Reagents and Tools Table, Materials and Methods, Figures, Data Availability Section)
Have **primary datasets** been deposited according to the journal's guidelines (see 'Data Deposition' section) and the respective accession numbers provided in the Data Availability Section?	Yes	Data availability section
Were **human clinical and genomic datasets** deposited in a public access-controlled repository in accordance to ethical obligations to the patients and to the applicable consent agreement?	Not Applicable	
Are **computational models** that are central and integral to a study available without restrictions in a machine-readable form? Were the relevant accession numbers or links provided?	Not Applicable	
If publicly available data were reused, provide the respective **data citations in the reference list**.	Not Applicable	
